# Predictors of disease severity in children presenting from the community with febrile illnesses: a systematic review of prognostic studies

**DOI:** 10.1136/bmjgh-2020-003451

**Published:** 2021-01-20

**Authors:** Arjun Chandna, Rainer Tan, Michael Carter, Ann Van Den Bruel, Jan Verbakel, Constantinos Koshiaris, Nahya Salim, Yoel Lubell, Paul Turner, Kristina Keitel

**Affiliations:** 1 Cambodia-Oxford Medical Research Unit, Angkor Hospital for Children, Siem Reap, Cambodia; 2 Centre for Tropical Medicine and Global Health, University of Oxford, Oxford, UK; 3 Unisanté Centre for Primary Care and Public Health, University of Lausanne, Lausanne, Switzerland; 4 University of Basel, Basel, Switzerland; 5 Swiss Tropical and Public Health Institute, Basel, Basel-Stadt, Switzerland; 6 Department of Women and Children's Health, King's College London, London, UK; 7 Academic Centre of General Practice, University of Leuven, Leuven, Flanders, Belgium; 8 Nuffield Department of Primary Care Health Sciences, University of Oxford, Oxford, UK; 9 Ifakara Health Institute, Dar-es-Salaam, Tanzania; 10 Department of Pediatrics and Child Health, Muhimbili University Health and Allied Sciences, Dar-es-Salaam, Tanzania; 11 Mahidol-Oxford Tropical Medicine Research Unit, Bangkok, Thailand; 12 Division of Emergency Medicine, Department of Pediatrics, University Children's Hospital, Inselpital, University of Bern, Bern, Switzerland

**Keywords:** paediatrics, child health, public health, infections, diseases, disorders, injuries, systematic review

## Abstract

**Introduction:**

Early identification of children at risk of severe febrile illness can optimise referral, admission and treatment decisions, particularly in resource-limited settings. We aimed to identify prognostic clinical and laboratory factors that predict progression to severe disease in febrile children presenting from the community.

**Methods:**

We systematically reviewed publications retrieved from MEDLINE, Web of Science and Embase between 31 May 1999 and 30 April 2020, supplemented by hand search of reference lists and consultation with an expert Technical Advisory Panel. Studies evaluating prognostic factors or clinical prediction models in children presenting from the community with febrile illnesses were eligible. The primary outcome was any objective measure of disease severity ascertained within 30 days of enrolment. We calculated unadjusted likelihood ratios (LRs) for comparison of prognostic factors, and compared clinical prediction models using the area under the receiver operating characteristic curves (AUROCs). Risk of bias and applicability of studies were assessed using the Prediction Model Risk of Bias Assessment Tool and the Quality In Prognosis Studies tool.

**Results:**

Of 5949 articles identified, 18 studies evaluating 200 prognostic factors and 25 clinical prediction models in 24 530 children were included. Heterogeneity between studies precluded formal meta-analysis. Malnutrition (positive LR range 1.56–11.13), hypoxia (2.10–8.11), altered consciousness (1.24–14.02), and markers of acidosis (1.36–7.71) and poor peripheral perfusion (1.78–17.38) were the most common predictors of severe disease. Clinical prediction model performance varied widely (AUROC range 0.49–0.97). Concerns regarding applicability were identified and most studies were at high risk of bias.

**Conclusions:**

Few studies address this important public health question. We identified prognostic factors from a wide range of geographic contexts that can help clinicians assess febrile children at risk of progressing to severe disease. Multicentre studies that include outpatients are required to explore generalisability and develop data-driven tools to support patient prioritisation and triage at the community level.

**PROSPERO registration number:**

CRD42019140542.

**Key questions**

**What is already known?**

- An increasing number of clinical decision-support algorithms and risk stratification tools integrate clinical and laboratory predictors to guide healthcare workers in their assessment of febrile children.
- Which prognostic factors—alone or as components of clinical prediction models—best identify children at risk of developing severe febrile illness is not clear.
- Previous systematic reviews have focused on diagnostic studies and used imperfect reference standards for severe disease.

**What are the new findings?**

- Malnutrition, hypoxia, altered consciousness, and bedside markers of acidosis and poor peripheral perfusion were the most commonly identified predictors of severe disease.
- Clinical prediction model performance varied—the best performing models being those evaluated in similar settings and using similar outcomes as the original derivation studies.
- The prognostic factors and clinical prediction models identified in this study reflect children with relatively advanced illnesses and hence the degree to which they can inform community triage and prioritisation strategies is unclear.

**Key questions**

**What do the new findings imply?**

- The studies included in this systematic review, together with other studies, highlight the importance of not over interpreting prognostic performance of individual predictors, which vary across different epidemiological contexts.
- If prediction models and decision-support algorithms are to be used as an adjunct to clinical assessment, they must be derived and validated using populations and outcomes appropriate to the clinical problem.
- To improve identification of children at risk of developing severe febrile illness, this will require multiple, large, collaborative research initiatives, which collect harmonised yet contextualised data on predictors and outcomes, and include unselected children presenting from the community.

## Introduction

Acute febrile illnesses are among the most common reasons that parents seek medical care for their children.^1 2^ While most episodes are mild, an important minority of children progress to severe disease. Early recognition of low-incidence serious disease is challenging,^3^ especially in many tropical settings where health workers receive limited training, patient volumes are high, diagnostic capacity is poor and different acute febrile syndromes are often clinically indistinguishable.^4 5^

Clinical and laboratory prognostic factors that enable early and accurate identification of children at risk of developing severe disease could improve patient outcomes and reduce resource misallocation.^6 7^ An increasing number of clinical decision-support algorithms and risk stratification tools integrate clinical and laboratory predictors to guide referral, admission and treatment decisions.^8^ While no unified strategy exists to guide selection of candidate predictors, those already reported as prognostic should normally be considered.^9^

Previous reviews have evaluated predictors of ‘serious bacterial infections’.^10 11^ However, these studies are diagnostic rather than prognostic.^9^ Furthermore, ‘serious bacterial infection’ is an imperfect measure of disease severity: microbiological tests for bacterial infections lack sensitivity, especially in settings with high antibiotic consumption; ‘serious bacterial infections’ are not always severe (eg, children with enteric fever are often successfully managed as outpatients) and severe febrile illnesses are frequently caused by non-bacterial pathogens, especially in low/middle-income countries (LMICs),^4 12^ in part secondary to the introduction of widespread vaccination against prevalent bacterial pathogens of childhood.^13^

We performed a systematic review to identify which clinical and laboratory factors—alone or as part of clinical prediction models—predict progression to severe disease in febrile children presenting from the community to a community health worker, primary health centre or hospital outpatient or emergency department. Our aim was to understand which prognostic factors might support health workers faced with this difficult and common clinical question and to inform variable selection for future prospective studies aiming to develop data-driven triage tools.

## Methods

### Protocol and registration

The methods for this systematic review were specified in advance (PROSPERO protocol: CRD42019140542) and adhere to the Checklist for Critical Appraisal and Data Extraction for Systematic Reviews of Prediction Modelling Studies (CHARMS),^14^ a modification of CHARMS for prognostic factor studies (CHARMS-PF),^15^ Quality In Prognosis Studies (QUIPS)^16^ and Prediction Model Risk of Bias Assessment Tool (PROBAST) guidelines.^17^ The report has been prepared in accordance with Preferred Reporting Items for Systematic Review and Meta-Analysis guidelines.^18^

### Eligibility criteria

All prognostic studies (prognostic factor and clinical prediction model) including ≥20 patients were eligible. Our target population was children aged >28 days and <19 years, presenting from the community with an acute febrile illness (documented abnormal temperature (fever or hypothermia) or history of fever) or suspected sepsis. While sepsis is not always well defined in children,^19^ ‘suspected sepsis’ was included along with febrile children so as to include all children with suspected infection. Studies were excluded if disaggregated paediatric data were not presented or patients were recruited partway through receipt of inpatient treatment, as the aim of the review was to identify *prognostic* variables measured at presentation. Studies that only evaluated specific clinical syndromes (eg, neurological presentations, acute respiratory infections and so on) or particular pathogens (eg, *Plasmodium* spp, influenza and so on) were not included.

Studies measuring predictors at presentation to care were included. Studies where authors identified that a substantial proportion of participants were recruited following transfer from another health facility were excluded. Demographic, anthropometric, socioeconomic, clinical and historical variables were considered, as well as laboratory parameters measured at presentation to care. Studies only reporting variables that would not be available at the time of presentation to care (eg, blood culture results) were excluded.

The primary outcome was any objective measure of disease severity occurring within 30 days of measurement of the predictors or during hospitalisation. Studies assessing outcome at the same time point as baseline predictor measurements (diagnostic studies) were excluded.

### Search strategy and selection criteria

We searched MEDLINE, Embase and Web of Science databases, without language restriction, for publications between 31 May 1999 and 30 April 2020 (initial search to 31 May 2019; updated search to 30 April 2020). We followed Cochrane Prognosis Methods Group recommendations to build our search strategy (online supplemental appendix S1), structured according to the ‘populations, interventions, comparators, outcomes, timing and setting’ (PICOTS) framework and adapted published search strings as appropriate.^20–22^ The search strategy was peer-reviewed by an independent Technical Advisory Panel (online supplemental appendix S2).

10.1136/bmjgh-2020-003451.supp1 Supplementary data

### Study selection

Title, abstract and full-text screening were performed independently by two reviewers (AC and RT). Agreement was checked after the first 20 and 250 articles. Discrepancies were resolved by discussion or independent assessment by a third reviewer (KK).

Eligible studies and relevant review articles were ‘snowballed’ (forward and reverse crosschecking of reference lists) to identify additional studies. The list of eligible studies was presented to the Technical Advisory Panel who were asked to identify obvious omissions and suggest key authors whose publication lists were subsequently reviewed for additional eligible studies (online supplemental appendix S2).

### Data collection process

Data extraction sheets were developed based on the CHARMS and CHARMS-PF checklists (online supplemental appendix S3).^14 15^ Data were extracted independently by one reviewer (AC or RT) and checked by the other. Discrepancies were discussed and resolved between the two reviewers. Authors of studies not reporting likelihood ratios (LRs) (prognostic factors) or area under the receiver operating characteristic curves (AUROCs) (clinical prediction models), or the data to allow their calculation, were contacted. Seven authors responded to requests for clarifications and six provided additional data not available in the published manuscript. All predictors were harmonised using the Systematised Nomenclature of Medicine Clinical Terms (SNOMED-CT).

### Data analysis: prognostic factors

Contingency tables were constructed and positive likelihood ratio (PLR) and negative likelihood ratio (NLR) calculated for each prognostic factor. In the case of an empty cell, 0.5 was added to each cell (Haldane-Anscombe correction). CIs were calculated on the basis of the SE of a proportion (Stata V.16.0). LRs were selected as the principal effect estimate as they allow estimation of post-test probabilities, are independent of prevalence, are intuitive for clinicians and are frequently used to compare performance of predictors in diagnostic and prognostic studies.^10 11 23 24^ Prognostic factors are presented in the main analysis if at least one study reported a PLR ≥5.0 (ie, a rule-in test), or a NLR ≤0.2 (ie, a rule-out test).^23^ To contextualise the results, we used the outcome prevalence of individual studies to calculate the pre-test probability, and display positive and negative post-test probabilities on dumbbell plots (R V.3.6.1).

### Data analysis: clinical prediction models

For clinical prediction models, AUROCs are presented on forest plots (Stata V.16.0). When available, we present LRs for different thresholds of the models in online supplemental appendix S4.

### Synthesis of results

Due to expected heterogeneity between studies (as a result of variations in case-mix and baseline risk), few common predictors for comparison and absence of well-defined subgroups, no formal meta-analysis nor comparison of variability and bias between studies was planned, as these comparisons are recognised as being prone to bias.^25^ Qualitative comparisons are described considering major differences between populations and study design. Prevalence of severe disease was used to group studies into low (<2.5%), moderate (2.5%–7.5%) and high (>7.5%) prevalence settings, as a proxy for the case-mix and level of care.

### Quality assessment

Risk of bias and applicability of studies were assessed using the QUIPS tool for prognostic factor studies,^16^ and PROBAST for studies developing, validating or updating prediction models.^17^ Each study was independently assessed using QUIPS or PROBAST by two reviewers (AC and RT), as well as an independent senior reviewer (MC, AVDB or JV). All discrepancies were resolved by discussion. For prognostic factor studies (QUIPS), risk of bias was categorised as low, medium or high, while in clinical prediction model studies (PROBAST) risk was categorised as low, high or unclear. For all studies, applicability was assessed as being of high, low or unclear concern.

### Role of the funding source

The funders had no role in study design, data collection, data analysis, data interpretation or writing of the report. The co-primary authors (AC and RT) had full access to the data and final responsibility for the decision to submit for publication.

### Patient and public involvement

Neither patients nor members of the public were directly involved in the conduct of this work.

## Results

**Figure 1:**
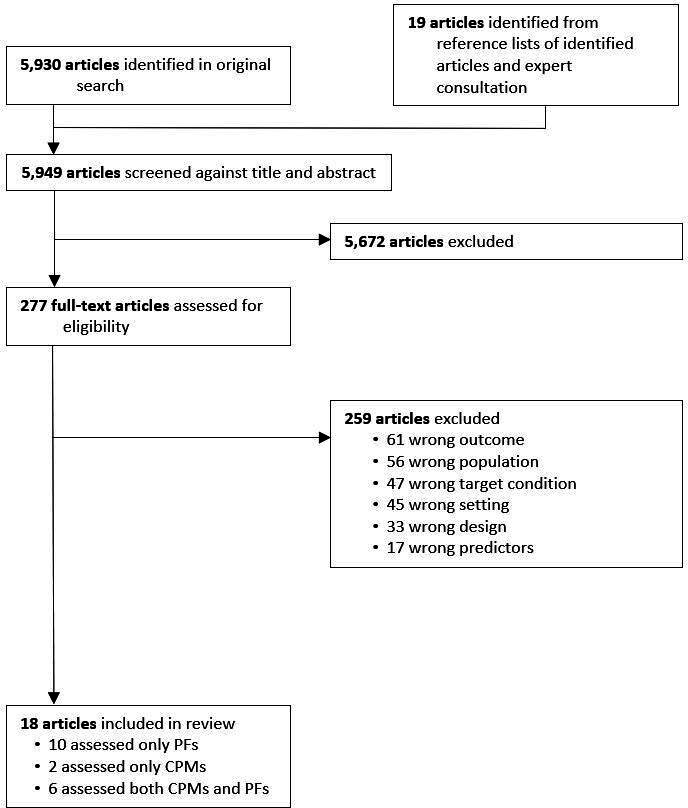
Selection of studies. Only one reason for exclusion per study is listed. CPM, clinical prediction model; PF, prognostic factor.

The electronic search retrieved 5930 articles, and 19 additional articles were identified through snowballing and expert consultation (figure 1). Eighteen studies were included in the review: 16 studies evaluated 200 prognostic factors, from 75 SNOMED-CT categories,^12 26–38^ and eight evaluated 33 clinical prediction model/outcome pairs, using 25 distinct models.^27 29 31 38–42^

In total 24 530 children were included, with overlap across eight studies.^26 31 32 34–37 40^ The majority (11/18) included only hospitalised patients. Two studies recruited children from primary care,^29 33^ and five recruited both children admitted and those sent home directly from hospital outpatient or emergency departments.^28 35–37 40^ Seven studies included children aged 5 years and under,^27 30 32–34 39 42^ with the remainder including patients up to 19 years of age. Definition of fever varied between studies, ranging from an axillary temperature (or equivalent) of ≥37.5°C to >38.1°C. Five studies did not include a temperature measurement in their eligibility criteria and enrolled children on the basis of suspected infection or sepsis.^35 38 40–42^ Eight studies were conducted in sub-Saharan Africa,^26 27 31–34 41 42^ four in North America,^35–37 40^ three in Europe,^29 30 38^ two in Asia^12 39^ and one in Latin America.^28^ Six were multicentre studies.^12 26 31 33 40 42^ Most used ‘hard’ outcomes to define severe disease, such as mortality, organ dysfunction or need for organ support, while four used ‘softer’ outcomes, such as prolonged length of stay or persistence of symptoms.^29 30 33 38^ Characteristics of the 18 studies are summarised in table 1.

**Table 1:** Characteristics of included studies

Study (year); setting, country	Cohort	Design	Quality assessment		CPM or PF study*	Sample size	Population		Outcome	Outcome prevalence (n/N)
			Risk of bias	Applicability concern			Inclusion criteria	Exclusion criteria		
**Outcomes including death, organ dysfunction, organ support and PICU admission**										
Scott (2020)^40^; Secondary and tertiary care hospitals, USA	Hospital OPD/ED	Retrospective cohort	High	Low	CPM	2464	Age 60 days to 18 years; Clinician-suspected sepsis	Hypotensive septic shock on arrival;† transfer to another centre; leaving ED before formal evaluation; incorrect registration	Hypotensive septic shock‡≤24 hour	11.4% (282/2464)
Walia (2016)^39^; Tertiary care hospital, India	Hospitalised§	Prospective cohort	High	High	CPM	100	Age 3–36 months; Axillary temperature >36.9°C (early morning) or >37.4°C	Non-infectious cause of fever; immunisation ≤2 days; immunodeficiency, autoimmune disorder	In-hospital mortality; Mechanical ventilation	11.0% (11/100); 17.0% (17/100)
Aramburo (2018)^26^; Secondary and tertiary care hospitals, Kenya, Tanzania and Uganda	Hospitalised§	Randomised controlled trial	Moderate	High	PF	3008	Age 60 days to 12 years; history of fever or axillary temperature ≥37.5°C or <36°C; severe febrile illness¶	Non-infectious cause of illness; SAM, gastroenteritis, burns, chronic kidney disease, pulmonary oedema, intoxication, surgical conditions, receipt of isotonic fluids during the same illness	In-hospital mortality (72 hours)	10.3% (309/3008)
George (2015)^31^; Secondary and tertiary care hospitals, Kenya, Tanzania and Uganda	Hospitalised§	Randomised controlled trial	High	High	CPM	3121	Age 60 days to 12 years; history of fever or axillary temperature ≥37.5°C or <36°C; severe febrile illness¶	Non-infectious cause of illness; SAM, gastroenteritis, burns, chronic kidney disease, pulmonary oedema, intoxication, surgical conditions, receipt of isotonic fluids during the same illness	In-hospital mortality (48 hours)	9.8% (306/3121)
Scott (2012)^37^; Tertiary care hospital, USA	Hospital OPD/ED	Prospective cohort	High	High	PF	239	Age <19 years; temperature >38.5°C or <36°C and heart rate >2 SD above normal for age; underwent phlebotomy as part of usual care	Transfer from another health facility; known inborn errors of metabolism; receipt of >15 min of intravenous therapy	24 hours organ dysfunction	5.4% (13/239)
Scott (2014)^36^; Tertiary care hospital, USA	Hospital OPD/ED	Prospective cohort	High	High	PF	239	Age <19 years; temperature >38.5°C or <36°C and heart rate >2 SD above normal for age; undergoing phlebotomy as part of routine care	Transfer from another health facility; known inborn errors of metabolism; receipt of >15 min of intravenous therapy	24 hours organ dysfunction	5.4% (13/239)
Nadjm (2013)^34^; Secondary care hospital, Tanzania	Hospitalised§	Prospective cohort	Moderate	High	PF	3319	Age 2 months to 5 years; history of fever in last 48 hours or axillary temperature ≥37.5°C	Chronic illness (excluding HIV and malnutrition); trauma; surgical conditions	In-hospital mortality	5.1% (170/3319)
Mtove (2011)^32^; Secondary care hospital, Tanzania	Hospitalised§	Prospective cohort	Moderate	High	PF	3248	Age 2 months to 13 years; history of fever in last 48 hours or axillary temperature ≥37.5°C	Chronic illness (excluding HIV and malnutrition); trauma; surgical conditions	In-hospital mortality	5.0% (164/3248)
Lowlaavar (2016)^42^; Secondary and tertiary care hospitals, Uganda	Hospitalised§	Prospective cohort	High	High	CPM	1307	Age 6–60 months; admitted during study working hours or within 8 hours of study shift with a proven or suspected infection	Previous enrolment; residence outside study catchment area	In-hospital mortality	5.0% (65/1307)
Conroy (2015)^27^; Tertiary care hospital, Uganda	Hospitalised§	Prospective cohort	High	High	CPM	2502	Age 2 months to 5 years; history of fever in last 48 hours or axillary temperature >37.5°C	None reported	In-hospital mortality	4.7% (99/2089)
van Nassau (2018)^38^; Secondary care hospital, The Netherlands	Hospitalised§	Retrospective cohort	High	High	CPM	864	Age <18 years; suspected bacterial infection**	Surgical conditions	PICU transfer and/or in-hospital mortality	2.7% (24/864)
Scott (2017)^35^; Tertiary care hospital, USA	Hospital OPD/ED	Retrospective cohort	Low	High	PF	1299	Age 60 days to 18 years; suspected sepsis††; measurement of venous lactate as part of routine care within 8 hours of ED arrival	Transfer from another health facility	30-day mortality	1.9% (25/1299)
SEAIDCRN (2017)^12^; Tertiary care hospitals, Indonesia, Thailand and Vietnam	Hospitalised§	Prospective cohort	High	High	PF	763	Age 30 days to 18 years; modified SIRS criteria‡‡	Suspicion of hospital-acquired infection; admission to hospital within previous 30 days; transfer from another health facility after >72 hours admission; weight <3 kg; enrolment in another clinical study	28-day mortality	1.9% (14/731)
Costa de Santana (2017)^28^; Tertiary care hospital, Brazil	Hospital OPD/ED	Retrospective cohort	High	High	PF	254	Age <13 years; axillary temperature >38.5°C; measurement of respiratory rate and heart rate on three occasions in absence of fever; measurement of leucocyte count as part of routine care	Congenital malformations; bronchopulmonary dysplasia; medullary aplasia; cardiac, renal or hepatic insufficiency	In-hospital mortality	1.6% (4/254)
Kwizera (2019)^41^; Secondary care hospital, Rwanda	Hospitalised§	Prospective cohort	High	High	CPM	949	Age 28 days to 18 years; confirmed acute infectious disease; symptom onset <14 days prior to hospital admission	Allergy to antimicrobials to treat sepsis (antibiotics, artesunate, artemether-lumefantrine); terminal disease	In-hospital mortality	1.5% (14/949)
**Outcomes including length of stay and persistence of symptoms**										
Freyne (2013)^30^; Secondary care hospital, Ireland	Hospitalised§	Prospective cohort	High	High	PF	46	Age 6–36 months; axillary temperature >38.1°C	Chronic illness; immunisation ≤2 days, antipyretic use ≤2 hours	Length of stay >96 hours	26.1% (12/46)
van Nassau (2018)^38^; Secondary care hospital, The Netherlands	Hospitalised§	Retrospective cohort	High	High	CPM	864	Age <18 years; suspected bacterial infection**	Surgical conditions	Length of stay ≥7 days	22.2% (179/806)
Elshout (2015)^29^; General Practice (out of hours), The Netherlands	Primary care	Prospective cohort	High	High	PF	480	Age 3 months to 6 years; history of fever	Communication in Dutch not possible; enrolment in last 2 weeks; direct referral to hospital required	Persistent fever at D3	13.1% (63/480)
Mwandama (2016)^33^; Community Health Workers, Malawi	Primary care	Prospective cohort	High	High	PF	285	Age 2–59 months; history of fever in last 48 hours or temperature ≥37.5°C; negative malaria rapid diagnostic test	Receipt of antimalarial in last 2 weeks; presence of danger signs§§	Persistent symptoms at D7	10.4% (19/182)

Studies are grouped according to the type of outcome they used: ‘hard’ (death, organ dysfunction, organ support, PICU admission) or ‘soft’ (length of stay, persistence of symptoms).

*Studies evaluating both PFs and CPMs were categorised on the basis of their primary analysis to facilitate review using the appropriate quality assessment tool.

†Hypotensive systolic blood pressure on arrival with receipt of a fluid bolus or vasoactive agent within 30 min.

‡Hypotension plus receipt of ≥30 mL/kg isotonic crystalloids or vasoactive medication.

§Only children the treating physician decided to admit were eligible but recruitment occurred at the time of admission to the health facility.

¶Respiratory distress (increased work of breathing or deep breathing) and/or impaired consciousness (coma or prostration) AND evidence of poor peripheral perfusion (capillary refill time >2 s or lower limb temperature gradient or weak radial pulse or severe tachycardia).

**Initiation of antibiotics within 24 hours of arrival in the emergency department.

††Decreased mental status or perfusion in the setting of suspected infection.

‡‡Rectal temperature >38.5°C or <35°C (or equivalent) AND heart rate >2 SD above normal for age (unless hypothermic) AND respiratory rate >2 SD above normal for age AND altered mental status OR systolic blood pressure <2 SD below normal for age OR pulse pressure <20 mm Hg OR capillary refill time >2 s OR SpO_2_ <95% OR leucocyte count >12×10^3^ cells/µL or <5×10^3^ cells/µL.

§§Convulsions, repeated vomiting, lethargy, severe anaemia or loss of consciousness.

CPM, clinical prediction model; ED, emergency department; n, number of outcomes; n, number of cases; OPD, outpatient department; PF, prognostic factor; PICU, paediatric intensive care unit; SAM, severe acute malnutrition; SEAIDCRN, Southeast Asia Infectious Disease Clinical Research Network; SIRS, systemic inflammatory response syndrome.;

### Prognostic factors

**Figure 2:**
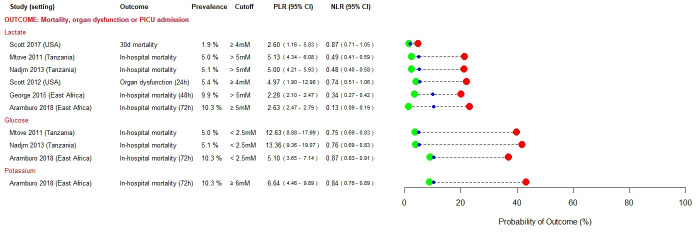
Prognostic factors identified as having rule-in (PLR ≥5.0) or rule-out (NLR ≤0.2) value for severe disease in at least one study—laboratory tests. mM, millimolar; NLR, negative likelihood ratio; PICU, paediatric intensive care unit; PLR, positive likelihood ratio.

**Figure 3:**
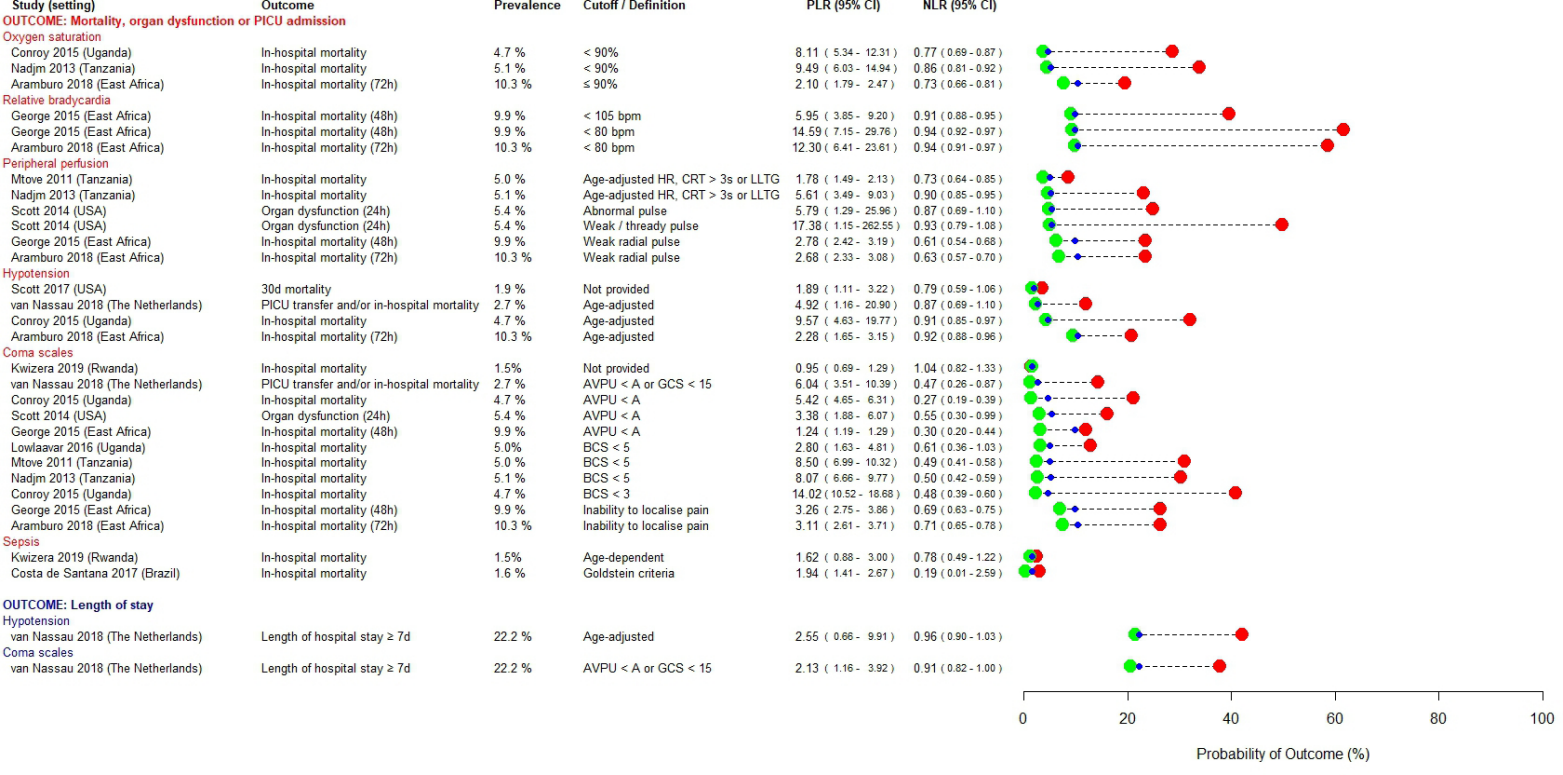
Prognostic factors identified as having rule-in (PLR ≥5.0) or rule-out (NLR ≤0.2) value for severe disease in at least one study—cardiovascular, respiratory or neurological signs. in the study by Costa *et al*. ‘sepsis’ was defined according to the systemic inflammatory response syndrome (SIRS), requiring measurement of heart rate, respiratory rate, temperature and leucocyte count. In the study by Kwizera *et al*, ‘sepsis’ was defined according to the qSOFA Score in children aged ≥15 years, and using a combination of temperature, mental status, respiratory distress, prostration and seizures in children aged <15 years. AVPU, alert, voice, pain or unresponsive; BCS, Blantyre Coma Score; bpm, beats per minute; CRT, capillary refill time; GCS, Glasgow Coma Score; HR, heart rate; LLTG, lower limb temperature gradient; NLR, negative likelihood ratio; PICU, paediatric intensive care unit; PLR, positive likelihood ratio; qSOFA, quick Sequential Organ Failure Assessment.

**Figure 4:**
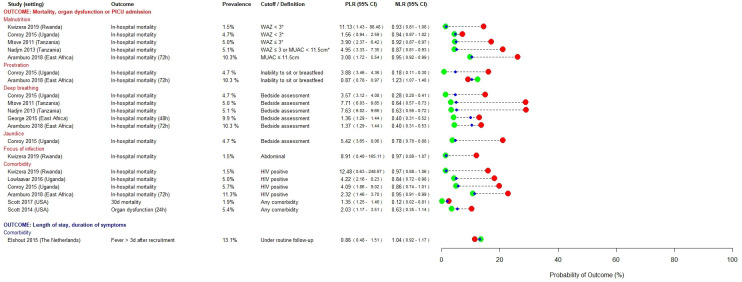
Prognostic factors identified as having rule-in (PLR ≥5.0) or rule-out (NLR ≤0.2) value for severe disease in at least one study—historical, anthropometric and metabolic variables. *Children with visible wasting or nutritional oedema were also classified as having severe malnutrition. In the study by Elshout *et al*, ‘comorbidity’ was defined as being under routine care of a paediatrician or ENT specialist. ENT, ear, nose and throat; MUAC, mid-upper arm circumference; NLR, negative likelihood ratio; PICU, paediatric intensive care unit; PLR, positive likelihood ratio; WAZ, weight-for-age z-score.

Figures 2–4 present prognostic factors identified as having rule-in (PLR ≥5.0) or rule-out (NLR ≤0.2) value in at least one study. Prognostic factors that met neither of these pre-specified cut-offs are presented in online supplemental appendix S5. In settings with moderate prevalence of severe disease, both high lactate (PLR range 4.97–5.13) and hypoglycaemia (PLR range 12.63–13.36) were useful for ruling in severe disease,^32 34 37^ whereas a lactate ≤5 mM was more useful as a rule-out test (NLR 0.13) among a population in whom prevalence of severe disease was high (febrile children with signs of poor organ perfusion).^26^

Hypoxia was most useful to rule-in severe disease in moderate prevalence settings (PLR range 8.11–9.49).^27 34^ Some studies found hypotension and bedside markers of poor peripheral perfusion to have useful rule-in value, but this was inconsistent (PLR range 1.89–9.57 and 1.78–17.38, respectively).^26 27 31 32 34–36 38^ Bradycardia was evaluated in a multicentre study conducted across three East African countries and found to have useful rule-in value (PLR range 5.95–14.59) for severe disease in those high prevalence settings.^26 31^ Impaired consciousness, assessed using bedside coma scales, was a useful predictor of severe disease, particularly in low and moderate prevalence settings (PLR range 3.38–14.02), with the post-test probability of poor outcome increasing with the degree of neurological impairment.^27 32 34 36 38 41 42^

In sub-Saharan African settings, severe malnutrition (PLR range 1.56–11.23),^26 27 32 34 41^ HIV positive status (PLR range 2.32–12.48)^26 27 41 42^ and bedside correlates of metabolic derangement such as deep breathing and jaundice (PLR range 3.57–7.71) were useful rule-in predictors, across a range of prevalence settings.^27 32 34^

Very few prognostic factors were satisfactorily able to rule-out progression to severe disease: presence of comorbidities (NLR range 0.12–1.04), sepsis at admission (NLR 0.19) and prostration (NLR range 0.18–1.23) were each identified in only one study.^27 28 35^

### Clinical prediction models

**Figure 5:**
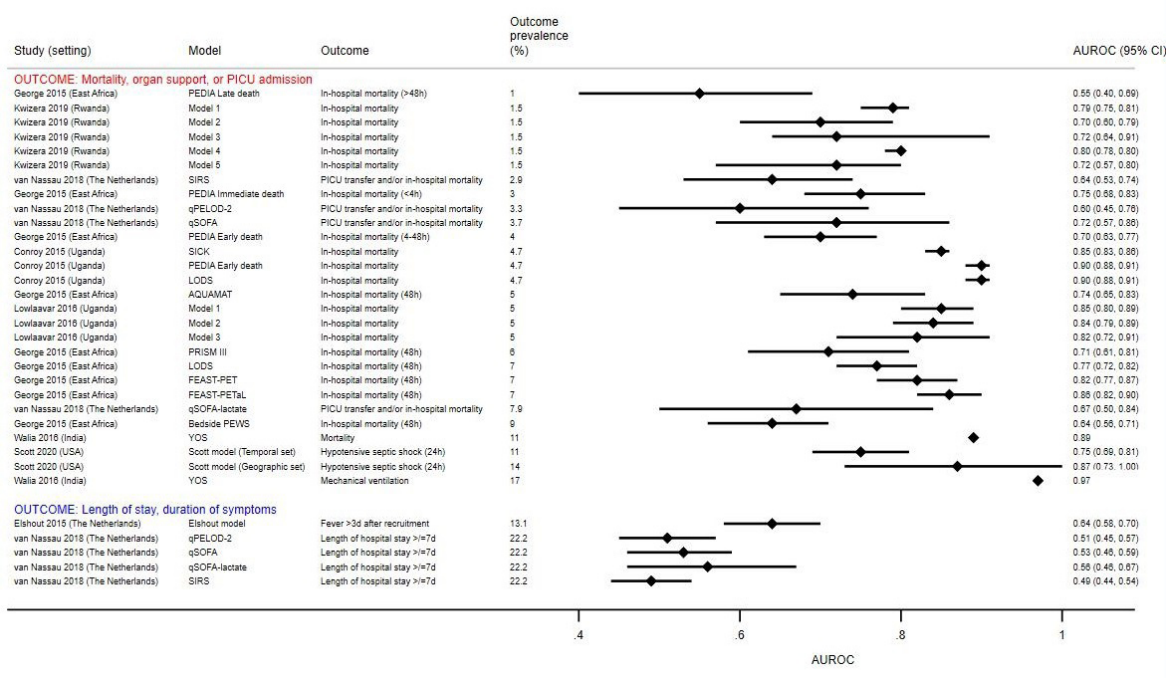
Discrimination of clinical prediction models to identify children at risk of severe disease. Individual studies evaluated different clinical prediction models using datasets with varying numbers of children with severe disease, depending on the data available. The outcome prevalence reflects the proportion of children with severe disease in the dataset used to evaluate that particular prediction model/outcome pair. This may be different from the overall prevalence of children with severe disease in the study, which is listed in table 1 and used to classify studies into low, moderate or high prevalence settings. No CIs were provided for the AUROC estimates in the study by Walia *et al*. AQUAMAT, African Quinine Artesunate Malaria Trial; AUROC, area under the receiver operating characteristic curve; FEAST-PET, FEAST-Paediatric Emergency Triage; FEAST-PETaL, FEAST-Paediatric Emergency Triage and Laboratory; LODS, Lambaréné Organ Dysfunction Score; PEDIA, Paediatric Early Death Index for Africa; PEWS, Paediatric Early Warning Score; PICU, paediatric intensive care unit; PRISM III, Paediatric Risk of Mortality; qPELOD-2, quick Paediatric Logistic Organ Dysfunction; qSOFA, quick Sequential Organ Failure Assessment; SICK, Signs of Inflammation in Children that Kill; SIRS, Systemic Inflammatory Response Syndrome; YOS, Yale Observation Score.

Figure 5 illustrates the discrimination (AUROC) of 25 clinical prediction models for 33 different outcomes assessed in eight studies: most (18/33) were external validations of existing models^27 31 38 39^; 13 were newly derived models^29 31 40–42^ and two were updates and external validations of an existing model.^38^ Components of the clinical prediction models are summarised in table 2.

**Table 2:** Components of clinical prediction models evaluated in the included studies

Clinical prediction model	Variables used in the clinical prediction model in the included studies	Included study evaluating the model	Original study developing the model
AQUAMAT	Base deficit, impaired consciousness, convulsions, elevated blood urea, underlying chronic illness	George^31^	von Seidlein^60^
ELSHOUT model	Sore throat, palpable lymph nodes, duration of fever before consultation, C-reactive protein	Elshout^29^	Elshout^29^
FEAST-PET	Axillary temperature, heart rate, capillary refill time, conscious level, respiratory distress, lung crepitations, severe pallor, weak pulse	George^31^	George^31^
FEAST-PETaL	FEAST-PET with the addition of lactate, pH, blood urea nitrogen	George^31^	George^31^
KWIZERA model 1	Age, respiratory rate, heart rate, temperature, capillary refill time, altered mental state	Kwizera^41^	Kwizera^41^
KWIZERA model 2	Age, respiratory rate, heart rate, capillary refill time, altered mental state	Kwizera^41^	Kwizera^41^
KWIZERA model 3	Age, respiratory rate, temperature, capillary refill time, altered mental state	Kwizera^41^	Kwizera^41^
KWIZERA model 4	Age, respiratory rate, capillary refill time, altered mental state	Kwizera^41^	Kwizera^41^
KWIZERA model 5	Age, respiratory rate, altered mental state	Kwizera^41^	Kwizera^41^
LODS	Deep breathing, coma, and prostration	George^31^; Conroy^27^	Helbok^50^
LOWLAAVAR model 1	Conscious level, HIV, weight-for-age z-score	Lowlaavar^42^	Lowlaavar^42^
LOWLAAVAR model 2	Conscious level, HIV, mid-upper arm circumference	Lowlaavar^42^	Lowlaavar^42^
LOWLAAVAR model 3	Conscious level, mid-upper arm circumference	Lowlaavar^42^	Lowlaavar^42^
PEDIA-i	Anaemia, jaundice, indrawing, deep breathing, conscious level, prostration, convulsions/seizures, temperature	George^31^	Berkley^51^
PEDIA-e	Jaundice, indrawing, conscious level, prostration, convulsions/seizures, wasting, kwashiorkor*	George;^31^ Conroy^27^	Berkley^51^
PEDIA-l	History >7 days, conscious level, prostration, convulsions/seizures, temperature, wasting, kwashiorkor	George^31^	Berkley^51^
PEWS†	Heart rate, capillary refill time, respiratory rate, oxygen saturation, systolic blood pressure	George^31^	Parshuram^61^
PRISM III‡	Heart rate, temperature, conscious level, systolic blood pressure, glucose, potassium, PCO_2_, pH, acidosis, pupillary reflexes	George^31^	Pollack^62^
qPELOD-2	Systolic or mean arterial pressure, heart rate, altered mentation	van Nassau^38^	Leclerc^53^
qSOFA	Respiratory rate, altered mentation, systolic blood pressure	van Nassau^38^	Seymour^54^
qSOFA-L	qSOFA with the addition of lactate	van Nassau^38^	van Nassau^38^
SCOTT model	Systolic blood pressure, diastolic blood pressure, temperature, age, respiratory rate, heart rate, arrival via emergency medical services, oncological comorbidity, indwelling central line on arrival, hospitalised in the last year	Scott^40^	Scott^40^
SICK	Level of consciousness, temperature, heart rate, respiratory rate, systolic blood pressure, SpO_2_, capillary refill time, age	Conroy^27^	Kumar^52^
SIRS	Heart rate, respiratory rate, leucocyte count, temperature	van Nassau^38^	Goldstein^63^
YOS	Quality of cry, reaction to parent stimulation, state variation, colour, hydration, response to social overtures	Walia^39^	McCarthy^64^

*Kwashiorkor was not included in the PEDIA-e score in the Conroy *et al* study.

†Receipt of oxygen therapy and respiratory effort included in the original PEWS but not measured in the George *et al* study.

‡Pupillary reflexes, pH, total CO_2_, arterial PaO_2_, creatinine, urea, white blood cells, prothrombin time and platelets included in the original PRISM III score but not measured in the George *et al* study.

AQUAMAT, African Quinine Artesunate Malaria Trial; FEAST-PET, FEAST-Paediatric Emergency Triage; FEAST-PETaL, FEAST-Paediatric Emergency Triage and Laboratory; LODS, Lambaréné Organ Dysfunction Score; PEDIA-e, Paediatric Early Death Index for Africa (early death score); PEDIA-i, Paediatric Early Death Index for Africa (immediate death score); PEDIA-l, Paediatric Early Death Index for Africa (late death score); PEWS, Paediatric Early Warning Score; PRISM-III, Paediatric Risk of Mortality; qPELOD-2, quick Paediatric Logistic Organ Dysfunction; qSOFA, quick Sequential Organ Failure Assessment; qSOFA-L, quick Sequential Organ Failure Assessment-Lacate; SICK, Signs of Inflammation in Children that Kill; SIRS, Systemic Inflammatory Response Syndrome; YOS, Yale Observation Score.

Three models, Lambaréné Organ Dysfunction Score (LODS), Paediatric Early Death Index for Africa (early death score) (PEDIA-e) and Signs of Inflammation in Children that Kill (SICK), showed good (AUROC ≥0.80) discrimination in a Ugandan setting where in-hospital mortality occurred at a prevalence of 4.7% (AUROC range 0.85–0.90).^27^ Two of these (LODS and PEDIA-e) were also assessed in a multicentre study in East Africa where discrimination was lower (AUROCs of 0.77 and 0.70).^31^ This study also derived two models, the FEAST-Paediatric Emergency Triage (FEAST-PET) and FEAST-Paediatric Emergency Triage and Laboratory (FEAST-PETaL) scores, which showed good discrimination (AUROCs of 0.86 and 0.82).^31^ Two other East African studies used combinations of simple clinico-demographic variables to derive a number of prediction models, four of which had AUROCs ≥0.80.^41 42^

One North American study derived a model to predict hypotensive shock in unselected children presenting with suspected sepsis, which showed good discrimination in an external geographic validation (AUROC 0.87).^40^ The Yale Observation Score also showed high discrimination for mortality (AUROC 0.97) and mechanical ventilation (AUROC 0.89) in India, however, the small sample size (n=100) renders the results difficult to interpret.^39^ In general, models assessed against ‘softer’ outcomes (eg, persistence of symptoms or length of stay) had poorer discrimination, and a more distal temporal relationship between measurement of predictors and ascertainment of outcome.

### Quality assessment

**Figure 6:**
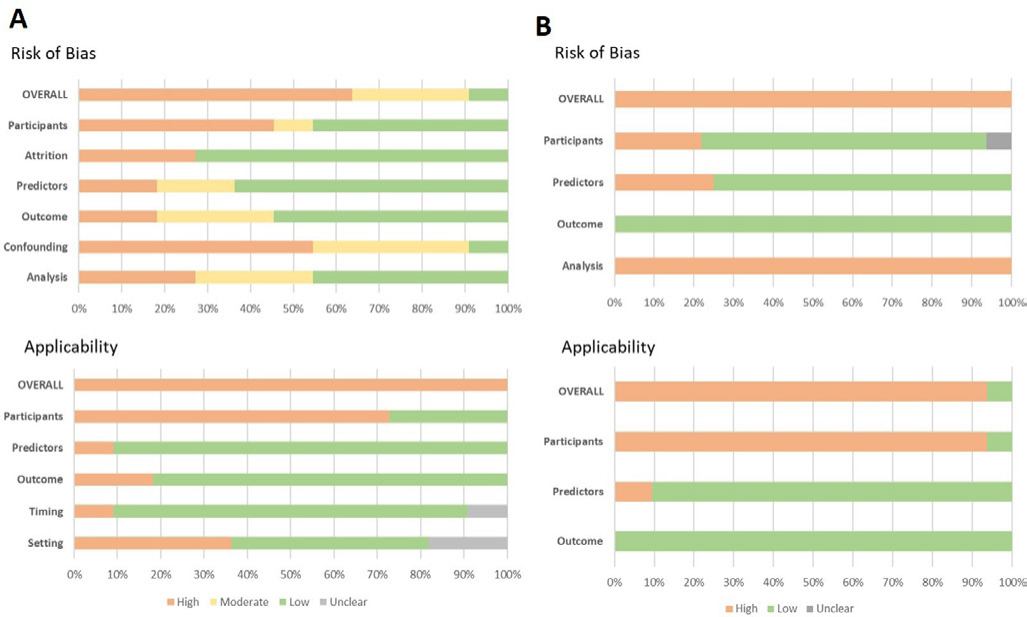
Risk of bias and applicability assessments for included studies using (A) the QUIPS tool (n=11 studies) and (B) PROBAST (n=33 clinical prediction model/outcome pairs from seven studies). All studies evaluating clinical prediction models were assessed using PROBAST, except for the study by Elshout *et al*, which was primarily a prognostic factor study and was therefore assessed using QUIPS. PROBAST, Prediction Model Risk of Bias Assessment Tool; QUIPS, Quality In Prognosis Studies.

Only one prognostic factor study was at low risk of bias,^35^ while another was judged to be at low risk of bias in all but one domain.^26^ The domains at highest risk of bias were study confounding, related to omission of important covariates; study participants, often due to requirement for the measurement of specific laboratory parameters (eg, leucocyte count); and statistical analysis, as a result of inadequate reporting or inappropriate exclusion of participants from the analysis (figure 6).

Each clinical prediction model/outcome pair was assessed independently and all judged to be at high risk of bias (figure 6). Most often this was due to inadequate reporting of model performance (studies reporting discrimination but not calibration), circularity between predictors and outcomes or having fewer than 100 participants with severe outcomes for model validation. It is noteworthy that one study which externally validated three models included 99 children who died.^27^ Another study which derived and/or validated nine models undertook an additional external validation in a population of acutely unwell but non-febrile children (and thus not eligible for consideration in this review), which included more than 100 children who died.^31^

In all but one study there was high concern regarding applicability to the review question.^40^ This was largely due to the majority of studies including only children requiring hospitalisation, with recruitment occurring after the decision to admit had been made by the treating physician. Full details on risk of bias and applicability assessments are provided in online supplemental appendix S6.

## Discussion

This systematic review of prognostic factors and clinical prediction models assessing severity of disease in febrile children highlights that few well-conducted studies address this important public health question, particularly in unselected children presenting from the community. One of its main strengths is the inclusion of studies from a wide geographic context, aiding understanding of how predictive performance can vary across settings. By focusing on prognosis, we identified features that predict the likelihood that a child’s illness might progress, rather than features associated with illness severity at the moment of assessment.

Most prognostic factors identified as valuable for predicting severe childhood febrile illness (PLR ≥5.0) overlapped with individual components of the most promising clinical prediction models (AUROC ≥0.80): nutritional and HIV status, hypoxia, altered consciousness, and markers of acidosis (raised venous lactate or deep breathing) and poor peripheral perfusion (weak pulse, limb-core temperature gradient or prolonged capillary refill time).^27 31 32 34 36 38 42^ Hypoglycaemia was a useful prognostic factor identified in our review, but omitted in most clinical prediction models. Many of these features, however, indicate a child that is already very unwell, reflecting the fact that most studies included only hospitalised children and focused on predicting mortality. Few prognostic factors adequately ruled-out (NLR ≤0.2) the possibility of progression to severe disease, a finding consistent with a previous systematic review evaluating the diagnostic utility of clinical features for serious bacterial infections.^10^

The major limitation of our work arises from the heterogeneity of studies, which precludes comparison of effect estimates. Second, it is difficult to determine if studies included children presenting to first-line health workers. We did not exclude studies solely based on the designated ‘level’ of a health facility: concerned parents in all settings use primary, secondary and tertiary care facilities as their first point-of-access. Third, most studies included only hospitalised children. This is a major barrier to understanding the potential for prognostic factors and prediction models to guide referral or admission decisions. Follow-up of children assessed as ‘low-risk’ (ie, those managed in the community) must be a priority for future studies seeking to determine the validity of prognostic factors and prediction models in outpatient settings.^43^ Fourth, in line with other reviews we found most studies to be of low quality.^44^ Recent guidance may help address this.^17^ Finally, we framed the review around ‘febrile illness’, rather than, for example, ‘clinically-suspected infection’. Our rationale was to ensure the findings were as relevant as possible for lesser-trained community health workers in resource-constrained settings, for whom a presumptive diagnosis of suspected infection can be challenging. Febrile illness is an accepted ‘pragmatic point-of-entry’ in these settings,^45^ however, we acknowledge that some children (particularly younger infants) may not mount a fever in response to serious infection. Therefore, despite our deliberately broad definition of febrile illness (documented abnormal temperature and history of fever), and the inclusion of studies of children with ‘suspected sepsis’, relevant studies may have been missed. Of note, in view of a suggestion arising during the peer-review process we also performed a second MEDLINE search, using alternate search strings, which did not yield any additional eligible articles (online supplemental appendix S7).

Thirty out of 200 (15%) prognostic factors met our pre-specified threshold for clinical relevance (PLR ≥5.0 or NLR ≤0.2). This may reflect the difficulty of identifying parsimonious predictors for all febrile children. While common pathophysiological pathways for severe disease have been identified across a spectrum of microbial aetiologies,^46 47^ certain predictors may perform better for specific syndromes or pathogens, compared with all-cause febrile illness. Five studies in our review reported a high proportion of children as being either slide-positive or rapid diagnostic test-positive for malaria. Notwithstanding the issues of co-infection and/or concomitant incidental parasitaemia in settings of high malaria endemicity, it is possible that the findings of these studies are more pertinent to children with malaria. However, four of these studies compared the prognostic performances of hyperlactaemia, hypoglycaemia and the prediction models SICK, LODS and PEDIA, and found them to be broadly consistent between children with malaria, non-malarial fever and invasive bacterial disease.^26 27 32 34^ Furthermore, as can be seen in figures 2–4, a number of predictors identified in malaria endemic regions also demonstrated prognostic utility in contexts where malaria is not endemic (eg, venous lactate, impaired peripheral perfusion, hypotension and altered consciousness). This, in conjunction with the subgroup analyses performed in the original studies, gives us a degree of confidence that the prognostic factors that we have identified are generalisable across different infecting pathogens. Nonetheless, future reviews using search strategies developed to retrieve syndrome-specific or pathogen-specific studies should explore this.

Another potential explanation for the relatively few valuable prognostic factors identified is work-up bias. In most studies, predictors were available to the treating clinicians: abnormal values are likely to have been acted on and predictive performance underestimated. For most predictors, particularly clinical signs, this is unavoidable as blinding is often neither possible nor ethical. When feasible, randomisation is required to definitively assess their potential impact.^48^ This is particularly important for new tests proposed in resource-limited settings. For example, both lactate and hypoxia were identified as potentially of value in this review but introducing tests for these parameters at all first-line health facilities across the tropics would incur substantial cost, and as their predictive value may vary in different settings, could result in unnecessary or missed referrals. Randomisation can help determine whether new tests such as these add value to simple clinical assessment.^49^

Clinical prediction models performed better when derived and validated in similar populations^27^: in East Africa LODS and PEDIA-e (both derived in sub-Saharan Africa)^50 51^ were superior to SICK (originally derived in India).^52^ Model performance also improved when predicting the same outcome as the derivation study: quick Sequential Organ Failure Assessment and quick Paediatric Logistic Organ Dysfunction, derived to predict mortality, performed poorly when predicting prolonged length of stay.^38 53 54^ These findings highlight the importance of deriving prediction models using populations and outcomes appropriate to the clinical question. While mortality is a ‘hard’ outcome, it seldom occurs in primary care. Furthermore, its reflection of disease severity is influenced (mediated) by the level of care. It is striking that in Tanzania a raised lactate conveyed a post-test probability of in-hospital mortality comparable to that of ‘organ dysfunction within 24 hours of arrival’ in a similar prevalence setting in the USA.^32 34 37^ Rather than relying on models derived in secondary care to generalise to outpatient settings across different epidemiological landscapes, alternative ways to quantify disease severity, which consider local context yet avoid circularity between predictor variables and outcome definitions, will be important to facilitate comparisons across settings and explore generalisability of risk prediction tools. Finally, the fact that most studies summarised model performance using only the AUROC means that is difficult to appreciate what the impact might be on clinical decision making.^55^

In LMIC primary care contexts, many variables are not feasible to collect,^56^ and as noted above, some may incur substantial cost. Interestingly, HIV and nutritional status were both identified in our review and represent the only prognostic factors meeting our threshold for clinical relevance that may not necessarily reflect a child that is overtly very unwell. While biological plausibility for the prognostic utility of these two variables is high, it should be noted that the study which identified them was small and correspondingly the CI for the PLR is wide.^41^ The WHO’s Integrated Management of Childhood Illnesses ‘Danger Signs’ are recommended to guide referrals from community healthcare providers in resource-constrained settings.^57^ Of these, only altered consciousness was widely represented among included studies, and most found it to be a good predictor of severe disease.^26 27 31 32 34 36 38 41 42^ History of convulsions was examined in two studies while other ‘Danger Signs’ were not evaluated.^26 27^

## Conclusion

Our findings emphasise the limitations of individual prognostic factors. Performance varies widely across settings and clinicians must be cognisant not to over interpret individual predictors. While prediction models can support clinical decision making, they must be derived and validated using appropriate methodology, and populations and outcomes relevant to the clinical problem. For the identification of children at risk of severe febrile illness, this will require multiple, large, collaborative, research initiatives across different settings, which collect harmonised data on predictors and outcomes,^58 59^ and include unselected children presenting from the community.

## Data Availability

Data are available upon request. All data relevant to the study are included in the article or uploaded as supplementary information. The protocol for the study is available from: https://www.crd.york.ac.uk/PROSPERO/display_record.php?RecordID=140542.

## References

[1] Prasad N, Sharples KJ, Murdoch DR, et al. Community prevalence of fever and relationship with malaria among infants and children in low-resource areas. Am J Trop Med Hyg 2015;93:178–80. 10.4269/ajtmh.14-0646 25918207 PMC4497891

[2] World Health Organization. WHO informal consultation on fever management in peripheral health care settings: a global review of evidence and practice. Geneva: World Health Organization, 2013.

[3] Buntinx F, Mant D, Van den Bruel A, et al. Dealing with low-incidence serious diseases in general practice. Br J Gen Pract 2011;61:43–6. 10.3399/bjgp11X548974 21401991 PMC3020049

[4] Prasad N, Murdoch DR, Reyburn H, et al. Etiology of severe febrile illness in low- and middle-income countries: a systematic review. PLoS One 2015;10:e0127962. 10.1371/journal.pone.0127962 26126200 PMC4488327

[5] Kruk ME, Gage AD, Joseph NT, et al. Mortality due to low-quality health systems in the universal health coverage era: a systematic analysis of amenable deaths in 137 countries. Lancet 2018;392:2203–12. 10.1016/S0140-6736(18)31668-4 30195398 PMC6238021

[6] McDonald CR, Weckman A, Richard-Greenblatt M, et al. Integrated fever management: disease severity markers to triage children with malaria and non-malarial febrile illness. Malar J 2018;17:353. 10.1186/s12936-018-2488-x 30305137 PMC6180660

[7] Molyneux E, Ahmad S, Robertson A. Improved triage and emergency care for children reduces inpatient mortality in a resource-constrained setting. Bull World Health Organ 2006;84:314–9. 10.2471/blt.04.019505 16628305 PMC2627321

[8] Keitel K, D'Acremont V. Electronic clinical decision algorithms for the integrated primary care management of febrile children in low-resource settings: review of existing tools. Clin Microbiol Infect 2018;24:845–55. 10.1016/j.cmi.2018.04.014 29684634

[9] Moons KGM, Altman DG, Reitsma JB, et al. Transparent reporting of a multivariable prediction model for individual prognosis or diagnosis (TRIPOD): explanation and elaboration. Ann Intern Med 2015;162:W1–73. 10.7326/M14-0698 25560730

[10] Van den Bruel A, Haj-Hassan T, Thompson M, et al. Diagnostic value of clinical features at presentation to identify serious infection in children in developed countries: a systematic review. Lancet 2010;375:834–45. 10.1016/S0140-6736(09)62000-6 20132979

[11] Van den Bruel A, Thompson MJ, Haj-Hassan T, et al. Diagnostic value of laboratory tests in identifying serious infections in febrile children: systematic review. BMJ 2011;342:d3082. 10.1136/bmj.d3082 21653621

[12] Southeast Asia Infectious Disease Clinical Research Network. Causes and outcomes of sepsis in Southeast Asia: a multinational multicentre cross-sectional study. Lancet Glob Health 2017;5:e157–67. 10.1016/S2214-109X(17)30007-4 28104185 PMC5332551

[13] Wahl B, O'Brien KL, Greenbaum A, et al. Burden of Streptococcus pneumoniae and Haemophilus influenzae type B disease in children in the era of conjugate vaccines: global, regional, and national estimates for 2000-15. Lancet Glob Health 2018;6:e744–57. 10.1016/S2214-109X(18)30247-X 29903376 PMC6005122

[14] Moons KGM, de Groot JAH, Bouwmeester W, et al. Critical appraisal and data extraction for systematic reviews of prediction modelling studies: the charms checklist. PLoS Med 2014;11:e1001744. 10.1371/journal.pmed.1001744 25314315 PMC4196729

[15] Riley RD, Moons KGM, Snell KIE, et al. A guide to systematic review and meta-analysis of prognostic factor studies. BMJ 2019;364:k4597. 10.1136/bmj.k4597 30700442

[16] Hayden JA, van der Windt DA, Cartwright JL, et al. Assessing bias in studies of prognostic factors. Ann Intern Med 2013;158:280–6. 10.7326/0003-4819-158-4-201302190-00009 23420236

[17] Moons KGM, Wolff RF, Riley RD, et al. PROBAST: a tool to assess risk of bias and applicability of prediction model studies: explanation and elaboration. Ann Intern Med 2019;170:W1–33. 10.7326/M18-1377 30596876

[18] Moher D, Liberati A, Tetzlaff J. Preferred reporting items for systematic reviews and meta-analyses: the PRISMA statement. Ann Intern Med 2009;151:264. **151**(4): 264-9, w64. 10.7326/0003-4819-151-4-200908180-00135 19622511

[19] Schlapbach LJ, Kissoon N. Defining pediatric sepsis. JAMA Pediatr 2018;172:313–4. 10.1001/jamapediatrics.2017.5208 29459982

[20] Geersing G-J, Bouwmeester W, Zuithoff P, et al. Search filters for finding prognostic and diagnostic prediction studies in MEDLINE to enhance systematic reviews. PLoS One 2012;7:e32844. 10.1371/journal.pone.0032844 22393453 PMC3290602

[21] Haynes RB, McKibbon KA, Wilczynski NL, et al. Optimal search strategies for retrieving scientifically strong studies of treatment from Medline: analytical survey. BMJ 2005;330:1179. 10.1136/bmj.38446.498542.8F 15894554 PMC558012

[22] Ingui BJ, Rogers MA. Searching for clinical prediction rules in MEDLINE. J Am Med Inform Assoc 2001;8:391–7. 10.1136/jamia.2001.0080391 11418546 PMC130084

[23] Jaeschke R, Guyatt GH, Sackett DL. Users' guides to the medical literature. III. How to use an article about a diagnostic test. B. what are the results and will they help me in caring for my patients? the evidence-based medicine Working group. JAMA 1994;271:703–7. 10.1001/jama.271.9.703 8309035

[24] Fischer JE, Bachmann LM, Jaeschke R. A readers' guide to the interpretation of diagnostic test properties: clinical example of sepsis. Intensive Care Med 2003;29:1043–51. 10.1007/s00134-003-1761-8 12734652

[25] Debray TPA, Damen JAAG, Snell KIE, et al. A guide to systematic review and meta-analysis of prediction model performance. BMJ 2017;356:i6460. 10.1136/bmj.i6460 28057641

[26] Aramburo A, Todd J, George EC, et al. Lactate clearance as a prognostic marker of mortality in severely ill febrile children in East Africa. BMC Med 2018;16:37. 10.1186/s12916-018-1014-x 29519240 PMC5844084

[27] Conroy AL, Hawkes M, Hayford K, et al. Prospective validation of pediatric disease severity scores to predict mortality in Ugandan children presenting with malaria and non-malaria febrile illness. Crit Care 2015;19:47. 10.1186/s13054-015-0773-4 25879892 PMC4339236

[28] Costa de Santana M. Duarte Mello Amoedo C, Nascimento-Carvalho CM. Clinical and epidemiological characteristics of children admitted with fever in emergency department with or without sepsis. J Infect Dev Ctries 2017;11:597–603. 31085820 10.3855/jidc.9257

[29] Elshout G, Kool M, Bohnen AM, et al. Predicting prolonged duration of fever in children: a cohort study in primary care. Br J Gen Pract 2015;65:e578–84. 10.3399/bjgp15X686485 26324494 PMC4540397

[30] Freyne B, Divilley R, Kissoon-Harrison G, et al. Field testing the utility of procalcitonin and the acute infantile observation score in febrile infants 6 to 36 months old presenting to the pediatric emergency department with no obvious focus of infection. Clin Pediatr 2013;52:503–6. 10.1177/0009922813483873 23613177

[31] George EC, Walker AS, Kiguli S, et al. Predicting mortality in sick African children: the feast paediatric emergency triage (PET) score. BMC Med 2015;13:174. 10.1186/s12916-015-0407-3 26228245 PMC4521500

[32] Mtove G, Nadjm B, Hendriksen ICE, et al. Point-Of-Care measurement of blood lactate in children admitted with febrile illness to an African district hospital. Clin Infect Dis 2011;53:548–54. 10.1093/cid/cir471 21865191

[33] Mwandama D, Mwale C, Bauleni A, et al. Clinical outcomes among febrile children aged 2 to 59 months with negative malaria rapid diagnostic test results in Mchinji district, Malawi. Malawi Med J 2016;28:150–3. 10.4314/mmj.v28i4.1 28321277 PMC5348606

[34] Nadjm B, Mtove G, Amos B, et al. Blood glucose as a predictor of mortality in children admitted to the hospital with febrile illness in Tanzania. Am J Trop Med Hyg 2013;89:232–7. 10.4269/ajtmh.13-0016 23817332 PMC3741242

[35] Scott HF, Brou L, Deakyne SJ, et al. Association between early lactate levels and 30-day mortality in clinically suspected sepsis in children. JAMA Pediatr 2017;171:249–55. 10.1001/jamapediatrics.2016.3681 28068437

[36] Scott HF, Donoghue AJ, Gaieski DF, et al. Effectiveness of physical exam signs for early detection of critical illness in pediatric systemic inflammatory response syndrome. BMC Emerg Med 2014;14:24. 10.1186/1471-227X-14-24 25407007 PMC4289256

[37] Scott HF, Donoghue AJ, Gaieski DF, et al. The utility of early lactate testing in undifferentiated pediatric systemic inflammatory response syndrome. Acad Emerg Med 2012;19:1276–80. 10.1111/acem.12014 23167859

[38] van Nassau SC, van Beek RH, Driessen GJ, et al. Translating Sepsis-3 criteria in children: prognostic accuracy of age-adjusted quick SOFA score in children visiting the emergency department with suspected bacterial infection. Front Pediatr 2018;6:266. 10.3389/fped.2018.00266 30327759 PMC6174358

[39] Walia S, M HM, Kumble A, et al. Yale observation scale as a predictor of bacteremia and final outcome in 3-36 months old febrile children admitted in tertiary health centres: a hospital-based cross-sectional study. Asian Journal of Pharmaceutical and Clinical Research 2016;9:219. 10.22159/ajpcr.2016.v9s3.11707

[40] Scott HF, Colborn KL, Sevick CJ, et al. Development and validation of a predictive model of the risk of pediatric septic shock using data known at the time of hospital arrival. J Pediatr 2020;217:145–51. 10.1016/j.jpeds.2019.09.079 31733815 PMC6980682

[41] Kwizera A, Kissoon N, Musa N, et al. A machine Learning-Based triage tool for children with acute infection in a low resource setting. Pediatr Crit Care Med 2019;20:1–30. 10.1097/PCC.0000000000002121 31805020

[42] Lowlaavar N, Larson CP, Kumbakumba E, et al. Pediatric in-hospital death from infectious disease in Uganda: derivation of clinical prediction models. PLoS One 2016;11:e0150683. 10.1371/journal.pone.0150683 26963914 PMC4786260

[43] Hansoti B, Jenson A, Keefe D, et al. Reliability and validity of pediatric triage tools evaluated in low resource settings: a systematic review. BMC Pediatr 2017;17:37. 10.1186/s12887-017-0796-x 28122537 PMC5267450

[44] Bouwmeester W, Zuithoff NPA, Mallett S, et al. Reporting and methods in clinical prediction research: a systematic review. PLoS Med 2012;9:e1001221. 10.1371/journal.pmed.1001221 PMC3358324 22629234

[45] World Health Organization. IMCI chart booklet. Geneva: World Health Organization, 2014.

[46] Leligdowicz A, Richard-Greenblatt M, Wright J, et al. Endothelial activation: the Ang/Tie axis in sepsis. Front Immunol 2018;9:838. 10.3389/fimmu.2018.00838 29740443 PMC5928262

[47] Kinasewitz GT, Yan SB, Basson B, et al. Universal changes in biomarkers of coagulation and inflammation occur in patients with severe sepsis, regardless of causative micro-organism [ISRCTN74215569]. Crit Care 2004;8:R82–90. 10.1186/cc2459 15025782 PMC420030

[48] Tan R, Kagoro F, Levine GA, et al. Clinical outcome of febrile Tanzanian children with severe malnutrition using anthropometry in comparison to clinical signs. Am J Trop Med Hyg 2020;102:427–35. 10.4269/ajtmh.19-0553 31802732 PMC7008344

[49] Keitel K, Samaka J, Masimba J, et al. Safety and efficacy of C-reactive Protein-guided antibiotic use to treat acute respiratory infections in Tanzanian children: a planned subgroup analysis of a randomized controlled Noninferiority trial evaluating a novel electronic clinical decision algorithm (ePOCT). Clin Infect Dis 2019;69:1926–34. 10.1093/cid/ciz080 30715250

[50] Helbok R, Kendjo E, Issifou S, et al. The Lambaréné organ dysfunction score (LODS) is a simple clinical predictor of fatal malaria in African children. J Infect Dis 2009;200:1834–41. 10.1086/648409 19911989

[51] Berkley JA, Ross A, Mwangi I, et al. Prognostic indicators of early and late death in children admitted to district hospital in Kenya: cohort study. BMJ 2003;326:361. 10.1136/bmj.326.7385.361 12586667 PMC148891

[52] Kumar N, Thomas N, Singhal D, et al. Triage score for severity of illness. Indian Pediatr 2003;40:204–10. 12657751

[53] Leclerc F, Duhamel A, Deken V, et al. Can the pediatric logistic organ Dysfunction-2 score on day 1 be used in clinical criteria for sepsis in children? Pediatr Crit Care Med 2017;18:758–63. 10.1097/PCC.0000000000001182 28492402

[54] Seymour CW, Liu VX, Iwashyna TJ, et al. Assessment of clinical criteria for sepsis: for the third International consensus definitions for sepsis and septic shock (Sepsis-3). JAMA 2016;315:762–74. 10.1001/jama.2016.0288 26903335 PMC5433435

[55] Fackler JC, Rehman M, Winslow RL. Please welcome the new team member: the algorithm. Pediatr Crit Care Med 2019;20:1200–1. 10.1097/PCC.0000000000002149 31804443

[56] Fung JST, Akech S, Kissoon N, et al. Determining predictors of sepsis at triage among children under 5 years of age in resource-limited settings: a modified Delphi process. PLoS One 2019;14:e0211274. 10.1371/journal.pone.0211274 30689660 PMC6349330

[57] World Health Organization. Integrated management of childhood Ilness chart booklet. Geneva: World Health Organization, 2014.

[58] Li E. Guidelines for the standardized collection of predictor variables in studies for pediatric sepsis. In: Ansermino M, ed. Scholars portal Dataverse. V2 ed, 2020.

[59] Wooldridge G, Murthy S, Kissoon N. Core outcome set in paediatric sepsis in low- and middle-income countries: a study protocol. BMJ Open 2020;10:e034960. 10.1136/bmjopen-2019-034960 PMC7245395 32265242

[60] von Seidlein L, Olaosebikan R, Hendriksen ICE, et al. Predicting the clinical outcome of severe falciparum malaria in African children: findings from a large randomized trial. Clin Infect Dis 2012;54:1080–90. 10.1093/cid/cis034 22412067 PMC3309889

[61] Parshuram CS, Hutchison J, Middaugh K. Development and initial validation of the bedside paediatric early warning system score. Crit Care 2009;13:R135. 10.1186/cc7998 19678924 PMC2750193

[62] Pollack MM, Patel KM, Ruttimann UE. Prism III: an updated pediatric risk of mortality score. Crit Care Med 1996;24:743–52. 10.1097/00003246-199605000-00004 8706448

[63] Goldstein B, Giroir B, Randolph A, et al. International pediatric sepsis consensus conference: definitions for sepsis and organ dysfunction in pediatrics. Pediatr Crit Care Med 2005;6:2–8. 10.1097/01.PCC.0000149131.72248.E6 15636651

[64] McCarthy PL, Sharpe MR, Spiesel SZ, et al. Observation scales to identify serious illness in febrile children. Pediatrics 1982;70:802–9. 7133831

