# Supplementary material for: Predictors of disease severity in children presenting from the community with febrile illnesses: a systematic review of prognostic studies
Source: BMJ Glob Health. 2021 Jan 20;6(1):e003451. doi: 10.1136/bmjgh-2020-003451 (PMC7818824; doi:10.1136/bmjgh-2020-003451)
Supplement: Supplementary data [file bmjgh-2020-003451supp001.pdf]

Predictors of disease severity in children presenting from the community with febrile illnesses: a systematic review of prognostic studies

Authors and affiliations

Arjun Chandna MD*,[1,2] Rainer Tan MD*,[3,4,5] Michael J Carter PhD,[6] Prof Ann Van den Bruel PhD,[7] Prof Jan Verbakel PhD,[7,8] Constantinos Koshiaris PhD,[8] Nahya Salim PhD,[9,10] Yoel Lubell PhD,[2,11] Paul Turner PhD**,[1,2] Kristina Keitel PhD**[4,12]

* Contributed equally
** Contributed equally

1. Cambodia Oxford Medical Research Unit, Angkor Hospital for Children, Cambodia
2. Centre for Tropical Medicine and Global Health, University of Oxford, UK
3. Unisanté, Centre for Primary Care and Public Health, University of Lausanne, Switzerland
4. Swiss Tropical and Public Health Institute (SwissTPH), Basel, Switzerland
5. University of Basel, Basel, Switzerland
6. Department of Women and Children's Health, King's College London, London, UK
7. Academic Centre of General Practice, University of Leuven, Leuven, Belgium
8. Nuffield Department of Primary Care Health Sciences, University of Oxford, Oxford, UK
9. Ifakara Health Institute, Dar es Salaam, United Republic of Tanzania
10. Department of Pediatrics and Child Health, Muhimbili University Health and Allied Sciences (MUHAS), Dar es Salaam, United Republic of Tanzania
11. Mahidol-Oxford Tropical Medicine Research Unit, Wellcome Trust Thailand Africa and Asia Programme
12. Division of Emergency Medicine, Department of Pediatrics, Inselspital Bern, Switzerland

Corresponding author

Dr. Arjun Chandna
Cambodia Oxford Medical Research Unit
Angkor Hospital for Children
Siem Reap
Cambodia
Email: arjun@tropmedres.ac
Tel: +855-85-712-586

Chandna A, *et al. BMJ Global Health* 2021; 6:e003451. doi: 10.1136/bmjgh-2020-003451

S1 Appendix

Table 1. Search strategy built following Cochrane Prognosis Methods Group recommendations[1] and adapting published search strings.[2-4]

	MEDLINE	Embase	Science Citation Index via Web of Science
**1**	Fever[MeSH Terms] OR Fever[Title/Abstract] OR Febrile[Title/Abstract] OR “suspected sepsis”[Title/Abstract]	fever/ or (fever* or febrile or suspected sepsis).ti,ab,kw.	TS=(fever* or febrile or "suspected sepsis")
**2**	pediatrics[MeSH Terms] OR pediatric*[Title/Abstract] OR paediatric*[Title/Abstract] OR child[MeSH Terms] OR child*[Title/Abstract] OR Infant[Mesh:NoExp] OR infant[Title/Abstract]	pediatrics/ or child/ or infant/ or preschool child/ or school child/ or toddler/ or boy/ or girl/ or (pediatric* or paediatric* or child* or infant*).mp.	TS=(pediatric* or paediatric* or child* or infant*)
**3**	(((((((Validat*[tw] OR Predict*[ti] OR Rule*[tw]) OR (Predict*[tw] AND (Outcome*[tw] OR Risk*[tw] OR Model*[tw])) OR ((History OR Variable*[tw] OR Criteria OR Scor*[tw] OR Characteristic*[tw] OR Finding*[tw] OR Factor*[tw]) AND (Predict*[tw] OR Model*[tw] OR Decision*[tw] OR Identif*[tw] OR Prognos*[tw])) OR (Decision*[tw] AND (Model*[tw] OR Clinical*[tw] OR “Logistic Models”[MeSH Terms])) OR (Prognostic AND (History OR Variable*[tw] OR Criteria OR Scor*[tw] OR Characteristic*[tw] OR Finding*[tw] OR Factor*[tw] OR Model*[tw])))) OR ("Stratification" OR "ROC Curve"[MeSH Terms] OR "Discrimination" OR "Discriminate" OR "c-statistic" OR "c statistic" OR "Area under the curve" OR "AUC" OR "Calibration" OR "Indices" OR "Algorithm" OR "Multivariable")))))	predict*.ti. or (validat* or rule* or (predict and (outcome* or risk* or model*)) or ((history or variable or criteria or scor* or characteristic* or finding* or factor*) and (predict* or model* or decision* or identif* or prognos*)) or (decision* and (model* or clinical*)) or (prognostic and (history or variable* or criteria or scor* or characteristic* or finding* or factor* or model*)) or stratification or discrimination or discriminate or c-statistic or "c statistic" or auc or calibration or indices or algorithm or multivariable).mp. or statistical model/ or "receiver operating characteristic"/ or "area under the curve"/	TI=(predict*) OR TS=(validat* or rule*) OR TS=((predict and (outcome* or risk* or model*))) OR TS=(((history or variable or criteria or scor* or characteristic* or finding* or factor*) and (predict* or model* or decision* or identif* or prognos*))) OR TS=((decision* and (model* or clinical*))) OR TS=((prognostic and (history or variable* or criteria or scor* or characteristic* or finding* or factor* or model*))) OR TS=(stratification or discrimination or discriminate or c-statistic or "c statistic" or auc or calibration or indices or algorithm or multivariable)
**4**	death[MeSH Terms] OR death[Title/Abstract] OR mortality[MeSH Terms] OR mortality[Title/Abstract] OR systemic inflammatory response syndrome[MeSH Terms] OR “systemic inflammatory response syndrome”[Title/Abstract] OR SIRS[Title/Abstract] OR sepsis[Title/Abstract] OR septic*[Title/Abstract] OR “severe disease*”[Title/Abstract] OR “severe infection*”[Title/Abstract] OR “severe bacterial infection*”[Title/Abstract] OR “severe illness”[Title/Abstract] OR “severe febrile illness”[Title/Abstract] OR “serious disease*”[Title/Abstract] OR “serious infection*”[Title/Abstract] OR “serious bacterial infection*”[Title/Abstract] OR “serious illness”[Title/Abstract] OR “serious febrile illness”[Title/Abstract]	mortality/ or childhood mortality/ or infant mortality/ or exp mortality rate/ or death/ or child death/ or fatality/ sepsis/ or systemic inflammatory response syndrome/ or exp septic shock/ or septicemia/ or (death or mortality or systemic inflammatory response or sirs or sepsis or septic* or ((severe or serious) adj2 (disease or illness* or infection*))).mp.	TS=(death or mortality or "systemic inflammatory response" or sirs or sepsis or septic*) OR TS=(((severe or serious) NEAR/2 (disease or illness* or infection*)))
**5**	1 AND 2 AND 3 AND 4	1 and 2 and 3 and 4	#4 AND #3 AND #2 AND #1
**6**	(“1999/05/31”[Date - Publication] : “2020/04/30”[Date – Publication])	conference*.pt.	#4 AND #3 AND #2 AND #1 Refined by: PUBLICATION YEARS: (2020 OR 2019 OR 2010 OR 2002 OR 2018 OR 2009 OR 2001 OR 2017 OR 2008 OR 2000 OR 2016 OR 2007 OR 1999 OR 2015 OR 2006 OR 2014 OR 2005 OR 2013 OR 2004 OR 2012 OR 2003 OR 2011)
**7**	5 AND 6	5 not 6	#4 AND #3 AND #2 AND #1 Refined by: PUBLICATION YEARS: (2020 OR 2019 OR 2010 OR 2002 OR 2018 OR 2009 OR 2001 OR 2017 OR 2008 OR 2000 OR 2016 OR 2007 OR 1999 OR 2015 OR 2006 OR 2014 OR 2005 OR 2013 OR 2004 OR 2012 OR 2003 OR 2011) AND [excluding] DOCUMENT TYPES: (MEETING ABSTRACT OR PROCEEDINGS PAPER)

S2 Appendix

Table 2. Membership of the Technical Advisory Panel (domain experts) responsible for peer-reviewing the search strategy, identifying omitted articles and suggesting key authors whose publication lists were reviewed.

<table>
<tr><th>Technical Advisory Panel member</th><th>Affiliation</th><th>Key authors proposed by Technical Advisory Panel</th></tr>
<tr><td>Dr. Jalemba Aluvaala</td><td>Paediatrics and Child Health, University of Nairobi, Nairobi, Kenya; KEMRI-Wellcome Trust Research Programme, Nairobi, Kenya</td><td rowspan="7">Ambrose Agweyu
Andre Siqueira
Anna Seale
Anthony Scott
Christopher C Moore
Climent Casals-Pascual
Elizabeth Molyneux
Henriette Moll
Kathryn Maitland
Jay Berkeley
Elizabeth Molyneux
Quique Bassat
Kristina E Rudd
Martin Otyek Opio
Michaëla A M Huson
Mike English
Mike Levin
Ruud Nijman
Samuel Akech
Tim Baker
Trevor Duke</td></tr>
<tr><td>Professor Quique Bassat</td><td>Centro de Investigação em Saúde de Manhiça, Maputo, Mozambique; ISGlobal, Hospital Clínic-Universitat de Barcelona, Barcelona, Spain; Institució Catalana de Recerca i Estudis Avançats, Barcelona, Spain.</td></tr>
<tr><td>Dr. David Bell</td><td>Foundation for Innovative New Diagnostics (FIND), Campus Biotech, Building B, Level 0, Chemin des Mines 9, 1202, Geneva, Switzerland.</td></tr>
<tr><td>Professor John Crump</td><td>Division of Infectious Diseases and International Health, Duke University Medical Center, Durham, North Carolina; Centre for International Health, University of Otago, Dunedin, New Zealand.</td></tr>
<tr><td>Professor W. Conrad Liles</td><td>Department of Medicine, University of Washington, Seattle, WA.</td></tr>
<tr><td>Dr. Rianne Oostenbrink</td><td>Department of General Paediatrics, Erasmus Medical Center Sophia Children's Hospital, Rotterdam, Netherlands.</td></tr>
<tr><td>Dr. Shunmay Yeung</td><td>Clinical Research Department, London School of Hygiene and Tropical Medicine, London, UK; Department of Paediatrics, Imperial College Healthcare NHS Trust, London, UK.</td></tr>
</table>

Chandna A, *et al. BMJ Global Health* 2021; 6:e003451. doi: 10.1136/bmjgh-2020-003451

S3 Appendix

Table 3. Data extraction sheet based on the CHARMS and CHARMS-PF checklists

Domain	Item	General	Applicability	Risk of bias	Extraction
**Study**	Study label	YES	NO	NO	
	Year of publication	YES	NO	NO	
	Journal of publication	YES	NO	NO	
	DOI	YES	NO	NO	
**Source of data**	Study design	YES	YES	YES	
	Target population	NO	YES	NO	
**Participants**	Single center or multi-center	YES	YES	YES	
	Number of centers recruiting	YES	NO	NO	
	Type of centers recruiting	YES	YES	YES	
	Location of study	YES	NO	NO	
	Recruitment method	YES	YES	YES	
	Recruitment setting	YES	YES	YES	
	Age range	YES	YES	YES	
	Fever definition + duration	YES	YES	YES	
	Inclusion criteria	YES	YES	YES	
	Exclusion criteria	YES	YES	YES	
	Participant description	YES	NO	NO	
	Study dates	YES	YES	NO	
**Outcomes to be predicted**	Prognostic outcome and definition	YES	YES	YES	
	Method of measurement of outcome	NO	NO	YES	
	Same outcome definition for all participants	NO	NO	YES	
	Same measurement of outcome for all participants	NO	NO	YES	
	Type of outcome (single or combined endpoints?)	YES	YES	NO	
	Outcomes assessed without knowledge of the candidate predictor (blinded)?	NO	NO	YES	
	Were candidate prognostic factors part of the outcome (e.g. when using a panel or consensus outcome measurement)?	NO	NO	YES	
	Time of outcome occurrence	YES	YES	NO	
**Prognostic factors**	Demographic prognostic factors	YES	NO	NO	
	Anthropometric prognostic factors	YES	NO	NO	
	Socioeconomic prognostic factors	YES	NO	NO	
	Historical (PMH) prognostic factors	YES	NO	NO	
	Clinical symptoms (current and historical during the illness) prognostic factors	YES	NO	NO	
	Clinical signs prognostic factors	YES	NO	NO	
	Vital signs prognostic factors	YES	NO	NO	
	Laboratory measures prognostic factors	YES	NO	NO	
	Score prognostic factors with definition and weights	YES	NO	NO	
	Method for measurement of prognostic factors	NO	NO	YES	

	Method of measurement of PFs is the same for all study participants?	NO	NO	YES	
	Setting of measurement of PF	YES	YES	YES	
	Setting of measurement of PF is the same for all study participants?	NO	NO	YES	
	Timing of prognostic factor measurement	NO	YES	YES	
	Prognostic factor assessed blinded for outcome?	NO	NO	YES	
	Handling of prognostic factor in the analysis (continuous, linear, categorised, non-linear transformations)	NO	NO	YES	
**Sample size**	Number of participants	YES	NO	NO	
	Number of refusals	NO	NO	YES	
	Number of outcomes/events	YES	NO	NO	
	For model studies: Number of outcomes/events in relation to the number of candidate prognostic factors (events per variable)	NO	NO	YES	
**Missing data**	Proportion of data on PF available for analysis	NO	NO	YES	
	Number of participants with missing data for each outcome	NO	NO	YES	
	Method used for missing data	NO	NO	YES	
**Analysis**	Modelling method (linear, logistic, cox, parametric survival, competing risks, regression)	YES	NO	YES	
	How modelling assumptions were checked (in particular, for time-to-event outcomes and the analysis of hazard ratios, the method for assessing non-proportional hazards (non-constant hazard ratios over time)	NO	NO	YES	
	Method for selection of PF for INCLUSION in multivariable modelling (all considered, preselection of established PF, retain only those significant from univariable analysis)	NO	NO	YES	
	Method for selection of PF DURING multivariable modelling	NO	NO	YES	
	Inclusion of additional PF (not measured at admission or not included in above categories) for multivariable modelling?	NO	YES	YES	
	Criteria used for any selection or exclusion of PF DURING multivariable modelling (P value, Akaike info criterion)	NO	NO	YES	
	Method of handling each continuous PF (dichotomisation, categorisation, linear, non-linear), including values of any cut-points used and their justification for non-linear relationships (splines, fractional polynomials)	NO	NO	YES	
**Results**	Unadjusted effect estimates for each PF	YES	NO	NO	
	Adjusted effect estimates for each PF	YES	NO	NO	
**Interpretation and discussion**	Interpretation of presented results	YES	YES	YES	
	Comparison with other studies	YES	YES	NO	
	Discussion of generalisability	YES	YES	NO	
	Strengths	YES	YES	YES	
	Limitations	YES	YES	YES	

Chandna A, et al. BMJ Global Health 2021; 6:e003451. doi: 10.1136/bmjgh-2020-003451

S4 Appendix

Table 4. Cut-points for clinical prediction models evaluated in the included studies associated with rule-in (positive likelihood ratio ≥ 5.0) or rule-out (negative likelihood ratio ≤ 0.2) value for progression to severe disease.

Model	Study	Outcome	Model score range	Cut-point to rule-in	PLR	NLR	Cut-point to rule-out	PLR	NLR
AQUAMAT	George 2015	In-hospital mortality (48h)	0-5	≥ 4	8.24	0.94			
FEAST-PET	George 2015	In-hospital mortality (48h)	0-10	≥ 6	7.95	0.54	≥ 3	1.36	0.10
LODS	George 2015	In-hospital mortality (48h)	0-3				≥ 1	1.13	0.00
LODS[a]	Conroy 2015	In-hospital mortality	0-3	> 1	6.49	0.21			
PEDIA-i	George 2015	In-hospital mortality (48h)	0-13	≥ 10	5.43	0.94	≥ 4	1.29	0.00
PEDIA-e	George 2015	In-hospital mortality (48h)	0-9	≥ 5	6.38	0.92	≥ 1	1.03	0.00
PEDIA-l	George 2015	In-hospital mortality (48h)	0-7	≥ 4	7.87	0.94	≥ 1	1.02	0.00
PEWS	George 2015	In-hospital mortality (48h)	0-19	≥ 15	5.66	0.97	≥ 1	1.00	0.00
PRISM III	George 2015	In-hospital mortality (48h)	0-24	≥ 6	5.70	0.58			
qPELOD-2[*]	van Nassau 2018	In-hospital mortality or PICU transfer	0-3	≥ 2	17.08	0.79			
qSOFA[*]	van Nassau 2018	In-hospital mortality or PICU transfer	0-3	≥ 2	7.46	0.54			
YOS	Walia 2016	Mortality	6-30	> 21	6.23	0.11	> 21[†]	6.23	0.11
YOS	Walia 2016	Mechanical ventilation	6-30	> 21	12.05	0.00	> 21[†]	12.05	0.00

NLR = negative likelihood ratio; PICU = pediatric intensive care unit; PLR = positive likelihood ratio

[*]Positive and negative likelihood ratios calculated from sensitivity and specificity provided in original manuscript; [†]For the Walia et al. study the same cut-point of > 21 was associated with a PLR ≥ 5.0 and NLR ≤ 0.2.

Chandna A, *et al. BMJ Global Health* 2021; 6:e003451. doi: 10.1136/bmjgh-2020-003451

S5 Appendix

Table 5a. Unadjusted likelihood ratios for prognostic factors judged to be of limited value (neither positive likelihood ratio ≥ 5.0 nor negative likelihood ratio ≤ 0.2 found in any study) to identify children at risk of progressing to severe febrile illness ('hard' outcomes).

Study	Cohort	Outcome	Prev.	Prognostic factor	Definition / Cut-off	PLR	95% CI	NLR	95% CI
**Demographic**									
Kwizera 2019	Hospitalised	In-hospital mortality	1.5	Age	< 12m	0.97	(0.27 – 3.52)	1.01	(0.81 – 1.25)
Kwizera 2019	Hospitalised	In-hospital mortality	1.5	Age	1y to < 5y	0.69	(0.34 – 1.40)	1.33	(0.90 – 1.98)
Kwizera 2019	Hospitalised	In-hospital mortality	1.5	Age	5y to < 12y	1.41	(0.69 – 2.87)	0.86	(0.58 – 1.27)
Kwizera 2019	Hospitalised	In-hospital mortality	1.5	Age	12y to < 18y	1.76	(0.48 – 6.46)	0.93	(0.75 – 1.16)
Scott 2017	Hospital OPD/ED	30-day mortality	1.9	Age	13y to 17y	0.57	(0.20 - 1.67)	1.11	(0.96 - 1.29)
Scott 2017	Hospital OPD/ED	30-day mortality	1.9	Age	6y to < 13y	1.21	(0.71 - 2.06)	0.91	(0.68 - 1.22)
Scott 2017	Hospital OPD/ED	30-day mortality	1.9	Age	12m to < 6y	1.15	(0.76 - 1.73)	0.90	(0.61 - 1.31)
Scott 2017	Hospital OPD/ED	30-day mortality	1.9	Age	< 12m	0.53	(0.08 - 3.66)	1.04	(0.96 - 1.13)
Conroy 2015	Hospitalised	In-hospital mortality	4.7	Age	< 12m	1.16	(0.88 - 1.52)	0.93	(0.80 - 1.08)
Mtove 2011	Hospitalised	In-hospital mortality	5.0	Age	< 12m	1.47	(1.22 - 1.78)	0.81	(0.71 - 0.92)
Nadjm 2013	Hospitalised	In-hospital mortality	5.1	Age	< 12m	1.42	(1.19 - 1.70)	0.81	(0.71 - 0.93)
Nadjm 2013	Hospitalised	In-hospital mortality	5.1	Age	< 24m	1.20	(1.10 - 1.30)	0.63	(0.47 - 0.83)
Aramburo 2018	Hospitalised	In-hospital mortality (72h)	10.3	Age	< 12m	1.47	(1.20 - 1.79)	0.90	(0.83 - 0.96)
Kwizera 2019	Hospitalised	In-hospital mortality	1.5	Sex	Female	0.87	(0.47 -1.60)	1.12	(0.88 – 1.06)
Scott 2017	Hospital OPD/ED	30-day mortality	1.9	Sex	Female	1.05	(0.67 - 1.64)	0.97	(0.68 - 1.37)
Conroy 2015	Hospitalised	In-hospital mortality	4.7	Sex	Female	0.97	(0.77 - 1.22)	1.02	(0.86 - 1.23)
Lowlaavar 2016	Hospitalised	In-hospital mortality	5.0	Sex	Female	1.02	(0.78 – 1.34)	0.98	(0.78 – 1.23)
George 2015	Hospitalised	In-hospital mortality (48h)	9.9	Sex	Female	1.01	(0.89 - 1.15)	0.99	(0.89 - 1.10)
Aramburo 2018	Hospitalised	In-hospital mortality (72h)	10.3	Sex	Female	1.04	(0.91 - 1.17)	0.97	(0.87 - 1.08)
**Anthropometric**									
George 2015	Hospitalised	In-hospital mortality (48h)	9.9	Weight	< 6kg	1.57	(0.92 - 2.68)	0.98	(0.96 - 1.01)
George 2015	Hospitalised	In-hospital mortality (48h)	9.9	Weight	< 8kg	1.29	(1.06 - 1.58)	0.92	(0.86 - 0.99)
**Historical**									
Scott 2017	Hospital OPD/ED	30-day mortality	1.9	Medical history	Non-oncological comorbidity	1.06	(0.68 - 1.66)	0.96	(0.67 - 1.36)
Scott 2017	Hospital OPD/ED	30-day mortality	1.9	Medical history	Oncological comorbidity	1.73	(1.17 - 2.54)	0.69	(0.46 - 1.03)
Scott 2014	Hospital OPD/ED	24-hour organ dysfunction	5.4	Medical history	Immunosuppressed	4.64	(1.79 - 12.00)	0.74	(0.52 - 1.07)

**Clinical symptoms**									
Conroy 2015	Hospitalised	In-hospital mortality	4.7	Convulsions	Caretaker history	1.90	(1.41 - 2.54)	0.81	(0.70 - 0.93)
Aramburo 2018	Hospitalised	In-hospital mortality (72h)	10.3	Convulsions	Caretaker history	1.39	(1.05 - 1.84)	0.95	(0.90 - 1.00)
George 2015	Hospitalised	In-hospital mortality (48h)	9.9	Fever	Caretaker history	1.00	(0.99 - 1.01)	1.42	(0.32 - 6.25)
Aramburo 2018	Hospitalised	In-hospital mortality (72h)	10.3	Urine looks dark	Caretaker history	1.04	(0.76 - 1.41)	0.99	(0.95 - 1.04)
**Clinical signs**									
van Nassau 2018	Hospitalised	PICU transfer and/or in-hospital mortality	2.7	Abnormal temperature	> 38.5°C or < 36°C	1.43	(0.93 - 2.20)	0.77	(0.50 - 1.17)
Conroy 2015	Hospitalised	In-hospital mortality	4.7	Hyperthermia	Temperature > 38°C	0.59	(0.42 - 0.82)	1.34	(1.18 - 1.51)
George 2015	Hospitalised	In-hospital mortality (48h)	9.9	Hyperthermia	Axillary temperature > 37°C	0.72	(0.66 - 0.80)	2.24	(1.92 - 2.62)
Aramburo 2018	Hospitalised	In-hospital mortality (72h)	10.3	Hyperthermia	Axillary temperature > 39°C	0.60	(0.45 - 0.79)	1.13	(1.07 - 1.19)
Conroy 2015	Hospitalised	In-hospital mortality	4.7	Hypothermia	Temperature < 36°C	3.16	(1.73 - 5.77)	0.92	(0.86 - 0.99)
Aramburo 2018	Hospitalised	In-hospital mortality (72h)	10.3	Hypothermia	Axillary temperature < 36°C	3.47	(2.58 - 4.67)	0.87	(0.83 - 0.92)
van Nassau 2018	Hospitalised	PICU transfer and/or in-hospital mortality	2.7	Heart rate	Age-adjusted	1.98	(1.33 - 2.94)	0.63	(0.40 - 0.99)
Conroy 2015	Hospitalised	In-hospital mortality	4.7	Heart rate	Age-adjusted	0.92	(0.77 - 1.09)	1.15	(0.90 - 1.46)
George 2015	Hospitalised	In-hospital mortality (48h)	9.9	Heart rate	≥ 200bpm	4.61	(1.99 - 10.67)	0.98	(0.96 - 1.00)
Aramburo 2018	Hospitalised	In-hospital mortality (72h)	10.3	Heart rate	Age-adjusted	0.74	(0.66 - 0.82)	1.69	(1.47 - 1.93)
Conroy 2015	Hospitalised	In-hospital mortality	4.7	Capillary refill time	≥ 3s	4.67	(3.00 - 7.28)	0.83	(0.75 - 0.92)
Scott 2014	Hospital OPD/ED	24-hour organ dysfunction	5.4	Capillary refill time	Flash or > 2s	0.50	(0.07 - 3.35)	1.09	(0.92 - 1.29)
George 2015	Hospitalised	In-hospital mortality (48h)	9.9	Capillary refill time	> 2s	1.28	(1.21 - 1.35)	0.48	(0.37 - 0.62)
Aramburo 2018	Hospitalised	In-hospital mortality (72h)	10.3	Capillary refill time	< 2s	0.50	(0.39 - 0.65)	1.26	(1.19 - 1.33)
Aramburo 2018	Hospitalised	In-hospital mortality (72h)	10.3	Capillary refill time	≥ 3s	1.98	(1.73 - 2.27)	0.69	(0.62 - 0.77)
Aramburo 2018	Hospitalised	In-hospital mortality (72h)	10.3	Capillary refill time	2-3s	0.84	(0.72 - 0.99)	1.11	(1.02 - 1.22)
Scott 2014	Hospital OPD/ED	24-hour organ dysfunction	5.4	Poor peripheral perfusion	Cold extremity	4.35	(0.52 - 36.17)	0.94	(0.80 - 1.10)
George 2015	Hospitalised	In-hospital mortality (48h)	9.9	Poor peripheral perfusion	Limb-core temp. gradient	1.34	(1.25 - 1.44)	0.54	(0.44 - 0.67)
Aramburo 2018	Hospitalised	In-hospital mortality (72h)	10.3	Poor peripheral perfusion	Limb-core temp. gradient	1.32	(1.23 - 1.42)	0.57	(0.47 - 0.70)
van Nassau 2018	Hospitalised	PICU transfer and/or in-hospital mortality	2.7	Respiratory rate	Age-adjusted	1.11	(0.93 - 1.33)	0.62	(0.22 - 1.78)
Conroy 2015	Hospitalised	In-hospital mortality	4.7	Respiratory rate	Age-adjusted	2.05	(1.66 - 2.53)	0.66	(0.54 - 0.80)
Conroy 2015	Hospitalised	In-hospital mortality	4.7	Respiratory distress	Subcostal recession	3.76	(3.18 - 4.45)	0.41	(0.31 - 0.54)
George 2015	Hospitalised	In-hospital mortality (48h)	9.9	Respiratory distress	Chest wall retraction	1.15	(1.07 - 1.23)	0.70	(0.56 - 0.86)
George 2015	Hospitalised	In-hospital mortality (48h)	9.9	Respiratory distress	Increased work of breathing or deep breathing	1.13	(1.09 - 1.17)	0.43	(0.29 - 0.63)
Aramburo 2018	Hospitalised	In-hospital mortality (72h)	10.3	Respiratory distress	Increased work of breathing or deep breathing	1.12	(1.08 - 1.16)	0.48	(0.34 - 0.69)

Chandna A, *et al. BMJ Global Health* 2021; 6:e003451. doi: 10.1136/bmjgh-2020-003451

George 2015	Hospitalised	In-hospital mortality (48h)	9.9	Respiratory crackles	Physician assessment	1.82	(1.54 - 2.14)	0.79	(0.72 - 0.86)
Aramburo 2018	Hospitalised	In-hospital mortality (72h)	10.3	Respiratory crackles	Physician assessment	1.81	(1.53 - 2.13)	0.80	(0.73 - 0.87)
Kwizera 2019	Hospitalised	In-hospital mortality	1.5	Focus of infection	Meningeal	2.71	(0.17 – 43.95)	0.98	(0.89 – 1.08)
Kwizera 2019	Hospitalised	In-hospital mortality	1.5	Focus of infection	Respiratory	0.50	(0.14 – 1.81)	1.20	(0.97 – 1.49)
Kwizera 2019	Hospitalised	In-hospital mortality	1.5	Focus of infection	Gastrointestinal	0.56	(0.08 – 3.71)	1.07	(0.92 – 1.23)
Kwizera 2019	Hospitalised	In-hospital mortality	1.5	Focus of infection	Urinary	1.69	(0.11-26.71)	0.99	(0.90 – 1.08)
Kwizera 2019	Hospitalised	In-hospital mortality	1.5	Focus of infection	Skin and/or soft-tissue	3.28	(0.20 -53.88)	0.98	(0.89 – 1.07)
George 2015	Hospitalised	In-hospital mortality (48h)	9.9	Cough	Physician assessment	1.00	(0.93 - 1.08)	1.00	(0.83 - 1.21)
Scott 2014	Hospital OPD/ED	24-hour organ dysfunction	5.4	Agitation	Physician assessment	4.17	(2.08 - 8.35)	0.61	(0.37 - 1.00)
George 2015	Hospitalised	In-hospital mortality (48h)	9.9	Seizures	Physician assessment	1.24	(0.96 - 1.61)	0.96	(0.91 - 1.01)
Aramburo 2018	Hospitalised	In-hospital mortality (72h)	10.3	Normal consciousness	AVPU = Alert (A)	0.29	(0.19 - 0.44)	1.23	(1.18 - 1.28)
George 2015	Hospitalised	In-hospital mortality (48h)	9.9	Vomiting	Physician assessment	1.14	(1.03 - 1.27)	0.85	(0.74 - 0.98)
George 2015	Hospitalised	In-hospital mortality (48h)	9.9	Dehydration	Decreased skin turgor	2.83	(2.07 - 3.89)	0.90	(0.86 - 0.95)
Aramburo 2018	Hospitalised	In-hospital mortality (72h)	10.3	Dehydration	Sunken eyes or reduced skin turgor	2.52	(1.89 - 3.36)	0.89	(0.85 - 0.94)
Aramburo 2018	Hospitalised	In-hospital mortality (72h)	10.3	Jaundice	Physician assessment	1.39	(1.21 - 1.60)	0.82	(0.75 - 0.91)
George 2015	Hospitalised	In-hospital mortality (48h)	9.9	Severe pallor	Physician assessment	1.47	(1.35 - 1.59)	0.56	(0.47 - 0.67)
Aramburo 2018	Hospitalised	In-hospital mortality (72h)	10.3	Severe pallor	Physician assessment	1.53	(1.37 - 1.71)	0.70	(0.62 - 0.80)
**Laboratory**									
Nadjm 2013	Hospitalised	In-hospital mortality	5.1	Glucose	> 5mmol/L	0.56	(0.47 - 0.67)	2.33	(2.02 - 2.68)
Nadjm 2013	Hospitalised	In-hospital mortality	5.1	Glucose	2.5-5mmol/L	1.39	(1.11 - 1.75)	0.88	(0.80 - 0.98)
Mtove 2011	Hospitalised	In-hospital mortality	5.0	Haemoglobin	< 4g/dL	1.98	(1.53 - 2.56)	0.83	(0.76 - 0.92)
Nadjm 2013	Hospitalised	In-hospital mortality	5.1	Haemoglobin	< 5g/dL	1.93	(1.51 - 2.46)	0.83	(0.75 - 0.92)
Aramburo 2018	Hospitalised	In-hospital mortality (72h)	10.3	Haemoglobin	< 5g/dL	1.43	(1.24 - 1.64)	0.81	(0.73 - 0.89)
Aramburo 2018	Hospitalised	In-hospital mortality (72h)	10.3	Haemoglobin	5-7g/dL	0.90	(0.68 - 1.19)	1.02	(0.97 - 1.07)
Aramburo 2018	Hospitalised	In-hospital mortality (72h)	10.3	Haemoglobin	7-10g/dL	0.97	(0.79 - 1.18)	1.01	(0.94 - 1.09)
Aramburo 2018	Hospitalised	In-hospital mortality (72h)	10.3	Haemoglobin	≥ 10g/dL	0.56	(0.42 - 0.75)	1.14	(1.09 - 1.20)
Aramburo 2018	Hospitalised	In-hospital mortality (72h)	10.3	Lactate	< 2.5mmol/L	0.26	(0.17 - 0.37)	1.35	(1.29 - 1.40)
Aramburo 2018	Hospitalised	In-hospital mortality (72h)	10.3	Lactate	2.5-5mmol/L	0.45	(0.34 - 0.58)	1.28	(1.21 - 1.35)
van Nassau 2018	Hospitalised	PICU transfer and/or in-hospital mortality	2.7	Leukocyte count	High or low (age-adjusted)	0.97	(0.64 - 1.48)	1.03	(0.67 - 1.58)
George 2015	Hospitalised	In-hospital mortality (48h)	9.9	pH	< 7.2	4.85	(3.79 - 6.21)	0.70	(0.63 - 0.77)
Aramburo 2018	Hospitalised	In-hospital mortality (72h)	10.3	pH	< 7.2	4.43	(3.45 - 5.68)	0.72	(0.65 - 0.79)
George 2015	Hospitalised	In-hospital mortality (48h)	9.9	Urea	> 20mg/dL	2.50	(2.08 - 3.00)	0.67	(0.58 - 0.76)

Aramburo 2018	Hospitalised	In-hospital mortality (72h)	10.3	Urea	> 20mg/dL	2.37	(1.97 - 2.84)	0.69	(0.61 - 0.78)
Aramburo 2018	Hospitalised	In-hospital mortality (72h)	10.3	Base deficit	> -8mmol/L	1.74	(1.61 - 1.88)	0.31	(0.23 - 0.43)
Aramburo 2018	Hospitalised	In-hospital mortality (72h)	10.3	Bicarbonate	< 15mmol/L	2.25	(2.03 - 2.51)	0.40	(0.31 - 0.50)
**Composite scores**									
Scott 2014	Hospital OPD/ED	24-hour organ dysfunction	5.4	CRS*	≥ 1	2.02	(1.26 - 3.23)	0.55	(0.28 - 1.11)
Scott 2014	Hospital OPD/ED	24-hour organ dysfunction	5.4	CRS*	≥ 2	4.35	(1.40 - 13.52)	0.81	(0.60 - 1.10)
SEAIDCRN 2017	Hospitalised	28-day mortality	1.9	Severe sepsis†	Goldstein criteria	3.08	(2.28 - 4.16)	0.29	(0.11 - 0.79)

AVPU = alert, voice, pain or unresponsive; CI = confidence interval; CRS = Clinical Recognition Signs; ED = emergency department; MUAC = mid-upper arm circumference; NLR = negative likelihood ratio; OPD = outpatient department; PLR = positive likelihood ratio; Prev. = outcome prevalence (%); WAZ = weight-for-age z-score

*CRS scored out of four variables including mental status, capillary refill time, peripheral pulse character, and presence of cold or mottled extremities);[5] †Children with sepsis were enrolled based on modified Goldstein criteria (see Table 1 in main manuscript). Severe sepsis was defined based on Goldstein criteria for severe sepsis.[6]

Chandna A, *et al. BMJ Global Health* 2021; 6:e003451. doi: 10.1136/bmjgh-2020-003451

Table 5b. Unadjusted likelihood ratios for prognostic factors judged to be of limited value (neither positive likelihood ratio ≥ 5.0 nor negative likelihood ratio ≤ 0.2 found in any study) to identify children at risk of progressing to severe febrile illness ('soft' outcomes).

Study	Cohort	Outcome	Prev.	Prognostic factor	Definition / Cut-off	PLR	95% CI	NLR	95% CI
**Demographic**									
Mwandama 2016	Primary care	Persistent symptoms at D7	10.4	Sex	Female	0.87	(0.53 - 1.42)	1.16	(0.73 - 1.83)
**Socioeconomic**									
Mwandama 2016	Primary care	Persistent symptoms at D7	10.4	Household socioeconomic status	Highest wealth quintile	1.24	(0.89 - 1.73)	0.71	(0.36 - 1.40)
Mwandama 2016	Primary care	Persistent symptoms at D7	10.4	Household socioeconomic status	Slept under ITN night prior to enrolment	0.73	(0.49 - 1.07)	2.08	(1.13 - 3.82)
Mwandama 2016	Primary care	Persistent symptoms at D7	10.4	Parental education	None	0.71	(0.10 - 5.20)	1.02	(0.91 - 1.15)
Mwandama 2016	Primary care	Persistent symptoms at D7	10.4	Parental education	Primary	1.05	(0.82 - 1.34)	0.86	(0.34 - 2.13)
Mwandama 2016	Primary care	Persistent symptoms at D7	10.4	Parental education	Secondary	0.92	(0.31 - 2.74)	1.02	(0.83 - 1.25)
**Clinical symptoms**									
Mwandama 2016	Primary care	Persistent symptoms at D7	10.4	URTI/cold presentation	Caretaker history	1.27	(0.81 - 2.00)	0.79	(0.46 - 1.35)
Elshout 2015	Primary care	Persistent fever at D3	13.1	Sore throat	Caretaker history	2.21	(1.39 - 3.51)	0.82	(0.70 - 0.96)
Elshout 2015	Primary care	Persistent fever at D3	13.1	Otalgia	Caretaker history	1.51	(0.92 - 2.47)	0.90	(0.78 - 1.05)
Elshout 2015	Primary care	Persistent fever at D3	13.1	Otalgia	Earache resulting in altered reaction or sleeping pattern	1.77	(0.85 - 3.68)	0.94	(0.85 - 1.04)
Elshout 2015	Primary care	Persistent fever at D3	13.1	Rhinorrhea	Caretaker history	1.19	(0.84 - 1.67)	0.91	(0.74 - 1.12)
Elshout 2015	Primary care	Persistent fever at D3	13.1	Cough	Caretaker history	1.20	(0.86 - 1.67)	0.90	(0.73 - 1.11)
Elshout 2015	Primary care	Persistent fever at D3	13.1	Moaning respiration	Caretaker history	1.27	(1.01 - 1.60)	0.77	(0.56 - 1.05)
Elshout 2015	Primary care	Persistent fever at D3	13.1	Abdominal pain	Caretaker history	1.45	(0.87 - 2.42)	0.92	(0.80 - 1.05)
Elshout 2015	Primary care	Persistent fever at D3	13.1	Diarrhea > 2/day	Caretaker history	1.45	(0.98 - 2.15)	0.87	(0.72 - 1.04)
Elshout 2015	Primary care	Persistent fever at D3	13.1	Vomiting	Caretaker history	1.31	(0.95 - 1.81)	0.85	(0.68 - 1.07)
Elshout 2015	Primary care	Persistent fever at D3	13.1	Febrile convulsions	Caretaker history	2.36	(1.04 - 5.35)	0.93	(0.85 - 1.02)
Elshout 2015	Primary care	Persistent fever at D3	13.1	Drowsy or difficult to wake	Caretaker history	0.85	(0.63 - 1.15)	1.15	(0.91 - 1.46)
Elshout 2015	Primary care	Persistent fever at D3	13.1	Restlessness or confusion	Caretaker history	1.09	(0.74 - 1.62)	0.96	(0.80 - 1.15)
Elshout 2015	Primary care	Persistent fever at D3	13.1	Feeling irritable	Caretaker history	1.31	(0.97 - 1.78)	0.84	(0.66 - 1.06)
Elshout 2015	Primary care	Persistent fever at D3	13.1	Inconsolable crying	Caretaker history	0.99	(0.75 - 1.32)	1.00	(0.79 - 1.28)
Elshout 2015	Primary care	Persistent fever at D3	13.1	Crying during diaper change	Caretaker history	0.95	(0.63 - 1.45)	1.02	(0.86 - 1.21)
Elshout 2015	Primary care	Persistent fever at D3	13.1	Crying when picked up	Caretaker history	0.86	(0.55 - 1.35)	1.06	(0.90 - 1.24)
Elshout 2015	Primary care	Persistent fever at D3	13.1	Different illness than usual	Caretaker history	1.16	(0.94 - 1.44)	0.81	(0.59 - 1.13)
Elshout 2015	Primary care	Persistent fever at D3	13.1	Parental concern	Caretaker history	1.51	(0.92 - 2.47)	0.90	(0.78 - 1.05)

Elshout 2015	Primary care	Persistent fever at D3	13.1	Drinking less than half usual	Caretaker history	1.07	(0.76 - 1.51)	0.96	(0.78 - 1.18)
Elshout 2015	Primary care	Persistent fever at D3	13.1	Skin rash	Caretaker history	0.92	(0.54 - 1.59)	1.02	(0.90 - 1.16)
Elshout 2015	Primary care	Persistent fever at D3	13.1	Pale, grey or spotted skin	Caretaker history	0.91	(0.69 - 1.21)	1.09	(0.85 - 1.39)
Elshout 2015	Primary care	Persistent fever at D3	13.1	Normal play behaviour	Caretaker history	1.09	(0.89 - 1.34)	0.87	(0.62 - 1.24)
Elshout 2015	Primary care	Persistent fever at D3	13.1	Normal reaction to parents	Caretaker history	0.70	(0.26 - 1.89)	1.03	(0.96 - 1.11)
**Clinical signs**									
Mwandama 2016	Primary care	Persistent symptoms at D7	10.4	Hyperthermia	Axillary temperature ≥ 37.5°C	1.64	(1.01 - 2.66)	0.67	(0.39 - 1.15)
Elshout 2015	Primary care	Persistent fever at D3	13.1	Hyperthermia	Rectal temperature ≥ 38°C	1.47	(1.08 - 2.01)	0.80	(0.63 - 1.00)
van Nassau 2018	Hospitalised	Length of stay >/= 7d	22.2	Abnormal temperature	> 38.5°C or < 36°C	0.81	(0.63 - 1.04)	1.11	(0.99 - 1.24)
van Nassau 2018	Hospitalised	Length of stay >/= 7d	22.2	Heart rate	Age-adjusted	1.67	(1.18 - 2.37)	0.88	(0.80 - 0.97)
Elshout 2015	Primary care	Persistent fever at D3	13.1	Capillary refill time	> 2s	0.98	(0.35 - 2.71)	1.00	(0.93 - 1.07)
van Nassau 2018	Hospitalised	Length of stay >/= 7d	22.2	Respiratory rate	Age-adjusted	0.99	(0.89 - 1.10)	1.04	(0.73 - 1.47)
Elshout 2015	Primary care	Persistent fever at D3	13.1	Respiratory distress (dyspnoea)	Physician assessment	1.06	(0.71 - 1.58)	0.98	(0.82 - 1.16)
Elshout 2015	Primary care	Persistent fever at D3	13.1	Pharyngitis	Sign of throat infection	1.64	(1.25 - 2.16)	0.70	(0.54 - 0.91)
Elshout 2015	Primary care	Persistent fever at D3	13.1	Rhinorrhoea	Physician assessment	0.91	(0.70 - 1.18)	1.11	(0.85 - 1.44)
Elshout 2015	Primary care	Persistent fever at D3	13.1	Cough	Physician assessment	1.28	(0.95 - 1.72)	0.84	(0.66 - 1.07)
Elshout 2015	Primary care	Persistent fever at D3	13.1	Palpable lymph nodes	Physician assessment	1.39	(1.09 - 1.77)	0.73	(0.54 - 0.98)
Elshout 2015	Primary care	Persistent fever at D3	13.1	Meningism	Able to put chin to chest	0.34	(0.05 - 2.47)	1.03	(0.99 - 1.07)
Elshout 2015	Primary care	Persistent fever at D3	13.1	Ill appearance	Physician assessment	1.32	(0.61 - 2.85)	0.97	(0.89 - 1.06)
**Laboratory**									
van Nassau 2018	Hospitalised	Length of stay >/= 7d	22.2	Leukocyte count	High or low (age-adjusted)	1.15	(0.96 - 1.36)	0.86	(0.70 - 1.06)
Freyne 2013	Hospitalised	Length of stay > 96h	26.1	Leukocyte count	> 15,000cells/mm	0.97	(0.44 - 2.15)	1.02	(0.57 - 1.82)
Freyne 2013	Hospitalised	Length of stay > 96h	26.1	Procalcitonin	> 1.0ng/L	1.00	(0.31 - 3.23)	1.00	(0.68 - 1.48)
Freyne 2013	Hospitalised	Length of stay > 96h	26.1	C-reactive protein	> 20mg/dL	1.27	(0.61 - 2.64)	0.82	(0.43 - 1.56)
**Composite scores**									
Freyne 2013	Hospitalised	Length of stay > 96h	26.1	AIOS*	> 10	1.00	(0.51 - 1.97)	1.00	(0.51 - 1.97)

AIOS = Acute Infantile Observation Score; CI = confidence interval; ITN = insecticide-treated bednet; NLR = negative likelihood ratio; PLR = positive likelihood ratio; Prev. = outcome prevalence (%)

*Acute Infantile Observation Score calculated as described for Yale Observation Score (YOS) in webappendix3 Table 3a.

Chandna A, et al. BMJ Global Health 2021; 6:e003451. doi: 10.1136/bmjgh-2020-003451

S6 Appendix

Table 6a: Risk of bias and applicability assessments for the included clinical prediction model studies (n=7) using PROBAST (Prediction model Risk Of Bias ASsessment Tool). Each prediction model/outcome pair (n=32) is assessed independently.

Study	Clinical prediction model	Outcome	Risk of Bias					Applicability			
			Overall	Analysis	Outcome	Predictors	Participants	Overall	Outcome	Predictors	Participants
George 2015	FEAST-PET (D)	In-hospital mortality (48h)	H	H	L	L	L	H	L	L	H
George 2015	FEAST-PETaL (D)	In-hospital mortality (48h)	H	H	L	L	L	H	L	L	H
George 2015	LODS (D)	In-hospital mortality (48h)	H	H	L	L	L	H	L	L	H
George 2015	PEDIA-i (V)	In-hospital mortality (<4h)	H	H	L	L	L	H	L	L	H
George 2015	PEDIA-e (V)	In-hospital mortality (4-48h)	H	H	L	L	L	H	L	L	H
George 2015	PEDIA-l (V)	In-hospital mortality (>48h)	H	H	L	L	L	H	L	L	H
George 2015	PRISM (V)	In-hospital mortality (48h)	H	H	L	L	L	H	L	L	H
George 2015	PEWS (V)	In-hospital mortality (48h)	H	H	L	L	L	H	L	L	H
George 2015	AQUAMAT (V)	In-hospital mortality (48h)	H	H	L	L	L	H	L	L	H
Conroy 2015	LODS (V)	In-hospital mortality	H	H	L	L	L	H	L	L	H
Conroy 2015	SICK (V)	In-hospital mortality	H	H	L	L	L	H	L	L	H
Conroy 2015	PEDIA-i (V)	In-hospital mortality	H	H	L	L	L	H	L	L	H
Lowlaavar 2016	Model 1 (D)	In-hospital mortality	H	H	L	H	L	H	L	H	H
Lowlaavar 2016	Model 2 (D)	In-hospital mortality	H	H	L	H	L	H	L	H	H
Lowlaavar 2016	Model 3 (D)	In-hospital mortality	H	H	L	H	L	H	L	H	H
Walia 2016	YOS (V)	Mortality	H	H	L	L	U	H	L	L	H
Walia 2016	YOS (V)	Mechanical ventilation	H	H	L	L	U	H	L	L	H
van Nassau 2018	qSOFA (V)	PICU transfer and/or in-hospital mortality	H	H	L	L	L	H	L	L	H
van Nassau 2018	qPELOD-2 (V)	PICU transfer and/or in-hospital mortality	H	H	L	L	L	H	L	L	H
van Nassau 2018	SIRS (V)	PICU transfer and/or in-hospital mortality	H	H	L	L	L	H	L	L	H
van Nassau 2018	qSOFA-L (V)	PICU transfer and/or in-hospital mortality	H	H	L	L	L	H	L	L	H
van Nassau 2018	qSOFA (V)	Length of stay ≥ 7 days	H	H	L	L	L	H	L	L	H
van Nassau 2018	qPELOD-2 (V)	Length of stay ≥ 7 days	H	H	L	L	L	H	L	L	H
van Nassau 2018	SIRS (V)	Length of stay ≥ 7 days	H	H	L	L	L	H	L	L	H

Chandna A, *et al. BMJ Global Health* 2021; 6:e003451. doi: 10.1136/bmjgh-2020-003451

van Nassau 2018	qSOFA-L (V)	Length of stay ≥ 7 days	H	H	L	L	L	H	L	L	H
Kwizera 2019	Model 1 (D)	In-hospital mortality	H	H	L	H	H	H	L	L	H
Kwizera 2019	Model 2 (D)	In-hospital mortality	H	H	L	H	H	H	L	L	H
Kwizera 2019	Model 3 (D)	In-hospital mortality	H	H	L	H	H	H	L	L	H
Kwizera 2019	Model 4 (D)	In-hospital mortality	H	H	L	H	H	H	L	L	H
Kwizera 2019	Model 5 (D)	In-hospital mortality	H	H	L	H	H	H	L	L	H
Scott 2020	Temporal (V)	Hypotensive shock ≤ 24h	H	H	H	L	H	L	L	L	L
Scott 2020	Geographic (V)	Hypotensive shock ≤ 24h	H	H	H	L	H	L	L	L	L

D = derivation; H = high risk/concern; L = low risk/concern; V = validation

Chandna A, *et al. BMJ Global Health* 2021; 6:e003451. doi: 10.1136/bmjgh-2020-003451

Table 6b. Risk of bias and applicability assessments for included prognostic factor studies (n=11) using the QUIPS (Quality in Prognosis Studies) tool.

Study ID	Risk of Bias							Applicability					
	Overall	Analysis	Confounding	Outcome	Predictors	Attrition	Participants	Overall	Setting	Timing	Outcome	Predictors	Participants
Elshout 2015	H	M	M	H	M	H	H	H	L	L	H	L	H
Scott 2012	H	L	H	M	L	L	H	H	L	H	L	L	H
Scott 2014	H	L	H	H	L	L	L	H	L	L	L	L	H
Scott 2017	L	L	L	L	L	L	L	H	L	L	L	L	H
Freyne 2013	H	H	H	M	L	L	H	H	U	L	L	L	H
Mtove 2011	M	L	M	L	L	L	L	H	H	L	L	L	L
Nadjm 2013	M	M	M	L	L	L	L	H	H	L	L	L	L
Aramburo 2018	M	L	M	L	L	L	L	H	H	L	L	L	H
Costa 2017	H	H	H	L	H	L	H	H	U	U	L	L	H
Mwandama 2016	H	M	H	M	M	H	H	H	L	L	H	L	H
SEAIDCRN 2017	H	H	H	L	H	H	M	H	H	L	L	H	L

H = high risk/concern; L = low risk/concern; U = unclear risk/concern

Chandna A, *et al. BMJ Global Health* 2021; 6:e003451. doi: 10.1136/bmjgh-2020-003451

S7 Appendix

Table 7. Alternate search strategy

	ORIGINAL MEDLINE SEARCH	ALTERNATE MEDLINE SEARCH
**1**	Fever[MeSH Terms] OR Fever[Title/Abstract] OR Febrile[Title/Abstract] OR “suspected sepsis”[Title/Abstract]	Fever[MeSH Terms] OR Fever[Title/Abstract] OR Febrile[Title/Abstract] OR “suspected sepsis”[Title/Abstract] OR Hypothermia[MeSH Terms] OR Hypothermia[Title/Abstract] OR “history of fever”[Title/Abstract]
**2**	pediatrics[MeSH Terms] OR pediatric*[Title/Abstract] OR paediatric*[Title/Abstract] OR child[MeSH Terms] OR child*[Title/Abstract] OR Infant[Mesh:NoExp] OR infant[Title/Abstract]	pediatrics[MeSH Terms] OR pediatric*[Title/Abstract] OR paediatric*[Title/Abstract] OR child[MeSH Terms] OR child*[Title/Abstract] OR Infant[Mesh:NoExp] OR infant[Title/Abstract]
**3**	(((((((Validat*[tw] OR Predict*[ti] OR Rule*[tw]) OR (Predict*[tw] AND (Outcome*[tw] OR Risk*[tw] OR Model*[tw])) OR ((History OR Variable*[tw] OR Criteria OR Scor*[tw] OR Characteristic*[tw] OR Finding*[tw] OR Factor*[tw]) AND (Predict*[tw] OR Model*[tw] OR Decision*[tw] OR Identif*[tw] OR Prognos*[tw])) OR (Decision*[tw] AND (Model*[tw] OR Clinical*[tw] OR “Logistic Models”[MeSH Terms])) OR (Prognostic AND (History OR Variable*[tw] OR Criteria OR Scor*[tw] OR Characteristic*[tw] OR Finding*[tw] OR Factor*[tw] OR Model*[tw])))) OR ("Stratification" OR "ROC Curve"[MeSH Terms] OR "Discrimination" OR "Discriminate" OR "c-statistic" OR "c statistic" OR "Area under the curve" OR "AUC" OR "Calibration" OR "Indices" OR "Algorithm" OR "Multivariable")))))	(((((((Validat*[tw] OR Predict*[ti] OR Rule*[tw]) OR (Predict*[tw] AND (Outcome*[tw] OR Risk*[tw] OR Model*[tw])) OR ((History OR Variable*[tw] OR Criteria OR Scor*[tw] OR Characteristic*[tw] OR Finding*[tw] OR Factor*[tw]) AND (Predict*[tw] OR Model*[tw] OR Decision*[tw] OR Identif*[tw] OR Prognos*[tw])) OR (Decision*[tw] AND (Model*[tw] OR Clinical*[tw] OR “Logistic Models”[MeSH Terms])) OR (Prognostic AND (History OR Variable*[tw] OR Criteria OR Scor*[tw] OR Characteristic*[tw] OR Finding*[tw] OR Factor*[tw] OR Model*[tw])))) OR ("Stratification" OR "ROC Curve"[MeSH Terms] OR "Discrimination" OR "Discriminate" OR "c-statistic" OR "c statistic" OR "Area under the curve" OR "AUC" OR "Calibration" OR "Indices" OR "Algorithm" OR "Multivariable")))))
**4**	death[MeSH Terms] OR death[Title/Abstract] OR mortality[MeSH Terms] OR mortality[Title/Abstract] OR systemic inflammatory response syndrome[MeSH Terms] OR “systemic inflammatory response syndrome”[Title/Abstract] OR SIRS[Title/Abstract] OR sepsis[Title/Abstract] OR septic*[Title/Abstract] OR “severe disease*”[Title/Abstract] OR “severe infection*”[Title/Abstract] OR “severe bacterial infection*”[Title/Abstract] OR “severe illness”[Title/Abstract] OR “severe febrile illness”[Title/Abstract] OR “serious disease*”[Title/Abstract] OR “serious infection*”[Title/Abstract] OR “serious bacterial infection*”[Title/Abstract] OR “serious illness”[Title/Abstract] OR “serious febrile illness”[Title/Abstract]	death[MeSH Terms] OR death[Title/Abstract] OR mortality[MeSH Terms] OR mortality[Title/Abstract] OR “severe disease*”[Title/Abstract] OR “severe infection*”[Title/Abstract] OR “severe bacterial infection*”[Title/Abstract] OR “severe illness”[Title/Abstract] OR “severe febrile illness”[Title/Abstract] OR “serious disease*”[Title/Abstract] OR “serious infection*”[Title/Abstract] OR “serious bacterial infection*”[Title/Abstract] OR “serious illness”[Title/Abstract] OR “serious febrile illness”[Title/Abstract]
**5**	1 AND 2 AND 3 AND 4	1 AND 2 AND 3 AND 4
**6**	(“1999/05/31”[Date - Publication] : “2020/04/30”[Date – Publication])	(“1999/05/31”[Date - Publication] : “2020/04/30”[Date – Publication])
**7**	5 AND 6	5 AND 6

Following suggestions arising during the peer review process we constructed an alternate search strategy which explicitly included the concept of ‘hypothermia’ and ‘history of fever’ in the first search string, and excluded the components of the third search string which were closely related to the concept of ‘suspected sepsis’. This search retrieved 2,470 articles on MEDLINE, 280 of which had not been retrieved by our original search. The Venn diagram below illustrates the overlap in studies retrieved by the two search strategies.

Two authors (AC and RT) independently screened the 280 additional articles against the eligibility criteria used for the systematic review: 279 were excluded by screening of title and abstract; one article proceeded to full text review but was subsequently excluded as 85% (306/360) of the cohort were neonates and data disaggregated by age were not presented. Hence, this alternate search strategy did not identify any additional eligible articles.

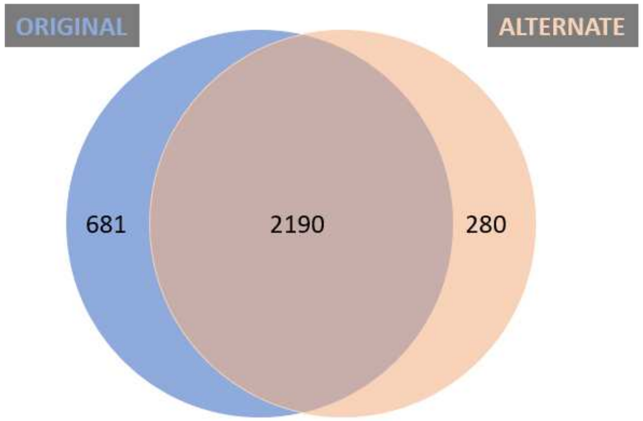

Figure 1. Venn diagram to illustrate the overlap in retrieved studies between the original and alternate search strategies.

References

1. Cochrane Prognosis Methods Group. Cochrane Methods Prognosis Tools. 2020. https://methods.cochrane.org/prognosis/tools (accessed 30 April 2020 2020).
2. Geersing GJ, Bouwmeester W, Zuithoff P, Spijker R, Leeflang M, Moons KG. Search filters for finding prognostic and diagnostic prediction studies in Medline to enhance systematic reviews. *PLoS One* 2012; **7**(2): e32844.
3. Haynes RB, McKibbon KA, Wilczynski NL, Walter SD, Werre SR. Optimal search strategies for retrieving scientifically strong studies of treatment from Medline: analytical survey. *Bmj* 2005; **330**(7501): 1179.
4. Ingui BJ, Rogers MA. Searching for clinical prediction rules in MEDLINE. *Journal of the American Medical Informatics Association : JAMIA* 2001; **8**(4): 391-7.
5. Scott HF, Donoghue AJ, Gaieski DF, Marchese RF, Mistry RD. Effectiveness of physical exam signs for early detection of critical illness in pediatric systemic inflammatory response syndrome. *BMC Emergency Medicine* 2014; **14**(1): 24.
6. Goldstein B, Giroir B, Randolph A. International pediatric sepsis consensus conference: definitions for sepsis and organ dysfunction in pediatrics. *Pediatric critical care medicine : a journal of the Society of Critical Care Medicine and the World Federation of Pediatric Intensive and Critical Care Societies* 2005; **6**(1): 2-8.
